# An overview of systematic reviews of complementary and alternative therapies for infantile colic

**DOI:** 10.1186/s13643-019-1191-5

**Published:** 2019-11-11

**Authors:** Rachel Perry, Verity Leach, Chris Penfold, Philippa Davies

**Affiliations:** 1 National Institute for Health Research Bristol Biomedical Research Centre, University Hospitals Bristol NHS Foundation Trust and University of Bristol, Nutrition Theme, 3rd Floor, Education & Research Centre, Upper Maudlin Street, Bristol, BS2 8AE UK; 2 Manchester Centre for Genomic Medicine, St Mary’s Hospital, Manchester University NHS Foundation Trust, Oxford Road, Manchester, UK; 3 The National Institute for Health Research Applied Research Collaboration (ARC), University Hospitals Bristol NHS Foundation Trust, Bristol, UK; 4 Population Health Sciences, Bristol Medical School, University of Bristol, Bristol, UK

**Keywords:** Colic, Systematic reviews, Overview, ROBIS, AMSTAR, Complementary and alternative medicine

## Abstract

**Background:**

Infantile colic is a distressing condition characterised by excessive crying in the first few months of life. The aim of this research was to update the synthesis of evidence of complementary and alternative medicine (CAM) research literature on infantile colic and establish what evidence is currently available.

**Methods:**

Medline, Embase and AMED (via Ovid), Web of Science and Central via Cochrane library were searched from their inception to September 2018. Google Scholar and OpenGrey were searched for grey literature and PROSPERO for ongoing reviews. Published systematic reviews that included randomised controlled trials (RCTs) of infants aged up to 1 year, diagnosed with infantile colic using standard diagnostic criteria, were eligible. Reviews of RCTs that assessed the effectiveness of any individual CAM therapy were included. Three reviewers were involved in data extraction and quality assessment using the AMSTAR-2 scale and risk of bias using the ROBIS tool.

**Results:**

Sixteen systematic reviews were identified. Probiotics, fennel extract and spinal manipulation show promise to alleviate symptoms of colic, although some concerns remain. Acupuncture and soy are currently not recommended. The majority of the reviews were assessed as having high or unclear risk of bias and low confidence in the findings.

**Conclusion:**

There is clearly a need for larger and more methodologically sound RCTs to be conducted on the effectiveness of some CAM therapies for IC. Particular focus on probiotics in non-breastfed infants is pertinent.

**Systematic review registration:**

PROSPERO: CRD42018092966.

## Background

### Description of the condition

Infantile colic (IC) is a common childhood condition affecting 5% to 20% of infants worldwide [1, 2]. In 2016, the new Rome IV criteria for colic defined it as ‘an infant who is less than five months of age when symptoms start and stop; recurrent and prolonged periods of infant crying, fussing or irritability reported by caregivers that occur without any obvious cause and cannot be prevented or resolved by caregivers; no evidence of infant failure to thrive, fever or illness [3]’. Prior to this, colic the was most commonly diagnosed using the Wessel ‘rule of three’ criteria [4].

Much research has been conducted over the past 50 years to try to establish the underlying aetiology. Formula intolerance, immaturity of gastrointestinal tract, food allergies, intestinal cramping or excessive gas formation have all been suggested [5], alongside psychosocial causes, e.g. maternal anxiety and maternal-infant bonding issues [6], but its pathophysiology remains unclear. Although it is considered a self-limiting condition, it can be distressing for both parents and babies.

#### Conventional treatment options

Our lack of understanding of IC makes it difficult to find an effective treatment. Current conventional treatments include dietary (particularly mother’s diet), physical, behavioural and pharmacological. With little evidence to support the first three approaches, the medication *Simethicone* is commonly used. However, this is no longer recommended on the NHS website [7].

#### CAM treatment options

Dissatisfaction with conventional health care and shortage of treatment options may lead parents to seek complementary and alternative (CAM) healthcare for their infants. CAM has been defined as ‘… diagnosis, treatment and/or prevention which complements mainstream medicine by contributing to a common whole, by satisfying a demand not met by orthodoxy or by diversifying the conceptual framework of medicine’ [8].

New parents in particular find IC stressful, resulting in high usage of CAM in this population [9]. Thus, further investigation is needed to evaluate the effectiveness and safety of CAM approaches and treatments. Information and advice regarding the treatment or management of colic is currently available to parents from a wide range of generally unregulated sources (e.g. websites) often which make claims that have no empirical support [10]. The main CAMs used for IC are probiotics, spinal manipulation, herbal medicine and acupuncture. A description of each alongside its justification for use in colic can be found in Additional file 1.

### Previous overview of reviews

There have been several previous overviews of reviews investigating CAM interventions for IC which have predominantly focused on spinal/chiropractic manipulation [11–13] or have included IC alongside other conditions affecting children [14]. Most lack adequate quality assessment of the included systematic reviews.

#### Why is it important to do this review?

Several trials have been published in recent years that indicate an effect of some CAMs for IC; these have mainly included trials of probiotics [6, 15–17]. The aim of this overview is to synthesise CAM research on IC and establish what evidence is currently available. As systematic reviews are considered the least biased source of evidence to evaluate the effectiveness of a particular intervention, this overview will focus on reviews of IC.

## Methods

This systematic overview was conducted following a pre-determined written protocol registered on the PROSPERO database: registration number CRD42018092966. To be considered eligible for this overview, reviews were required to meet the following criteria:

Type of reviews—all systematic reviews of randomised controlled trials (RCTS) of infantile colic (IC).

Type of participants—human subjects diagnosed with infantile colic using standard diagnostic criteria (e.g. WESSEL criteria^3^). No restrictions regarding gender, condition duration or intensity were applied. Age was restricted to infants under 1 year.

Type of intervention—reviews of effects of any CAM therapies. Reviews that included multiple CAM therapies were also included, as long as the CAM therapies were not used in combination.

Type of comparator—placebo, active treatment, no treatment, treatment-as-usual or waitlist control groups.

Type of outcome—any review that included studies that reported measures of colic severity (e.g. parent-reported crying diaries; questionnaires and parental interviews).

The full criteria are listed in Table S4, Additional file 1.

### Data sources

Medline, Embase and AMED (via Ovid), Web of Science and Central via Cochrane library were searched from their inception to September 2018. Google Scholar (first 20 pages) and OpenGrey were searched for grey literature and PROSPERO for ongoing reviews. All reviews from 2011 were assessed for eligibility. The search strategy was structured using subject (MeSH) headings, text word terms and their derivatives: homoeopath, acupuncture, spinal manipulation, hypnosis, reflexology, phytotherapy, probiotics, infant, colic, systematic review, meta-analysis. Full details of the search can be found in Additional file 1.

### Data extraction

One reviewer (RP) extracted data and summarised the review characteristics (see Table 1). Extracted data was checked by another reviewer (VL or PD). Disagreements were resolved through discussion. Information was extracted on author, date of review, country, list of studies included in the individual review, intervention and comparator summary, number of participants, diagnosis criteria, meta-analysis results or summary of main between-group results, whether a sensitivity or subgroup analysis was conducted, risk of bias assessment and adverse events.

**Table 1:** Summary of the included reviews

Author (date) country	Inclusion criteria	Details of search	CAM of interest	Primary outcome	Meta-analysis: Y/N Subgroup/sensitivity analysis: Y/N	Risk of bias assessment: safety/adverse events mentioned	Conclusions (irrelevant information removed)
Multiple CAM therapies							
Perry UK [22]	RCTs, children diagnosed with IC (e.g. Wessel), any form of CAM versus placebo, no treatment, TAU or WL as control groups	5 databases from inception to February 2010 No language/date restrictions Grey literature not searched	Supplements, herbal, massage, reflexology, manipulation, mixed treatment	RCTs with the following primary outcomes: subjective measures of colic severity in parental self- report/observer completed QoL parameters; in physiologic parameters; reduction in the need for medication, other treatment or adverse effects of treatment (from BL)	No: No	Jadad score: Yes	Some encouraging results exist for fennel extract, mixed herbal tea and sugar solutions, although all trials have major limitations. Thus, the notion that any form of CAM is effective for infantile colic currently is not supported from the evidence from the included RCTs. Additional replications are needed before firm conclusions can be drawn.
Bruyas-Bertholon [23] France	RCTs or MAs Therapeutic evaluation of colic or excessive crying in infants < 6 mths	3 databases to December 2010 French and English papers only Grey literature not searched	Non-allopathic drug, manual therapies, soy	NR	No: No	Jadad score: Some trials reported AEs	The main therapeutic strategies currently validated are, fennel herbal medicine and the probiotic *L*. *reuteri*. It seems reasonable to combine reassurance and healthy lifestyle counselling with parents, and a limitation of stimulation around the child. These results could serve as a basis for a consensus conference on the management of infant colic.
Harb [24] Australia	RCTs (incl. crossover), published after 1 January 1980 The ppts were mothers of colicky fully or partially breast-fed infants < 6 mths Wessel (incl. modified) Excluded if the sample size was < 16 ppts	5 databases searched from July 2014?? to 31 July, 2015 Grey literature not searched	Probiotic/symbiotic, phytotherapies (NB: other therapies included but not relevant to our report)	Changes in crying duration, response rates as measured by a reduction in symptoms	Yes: Yes	Cochrane RoB: No	Probiotics, in particular, *L*. *reuteri*, and preparations containing fennel oil appear effective for reducing colic, although there are limitations to these findings. The evidence for sucrose, glucose, is weak. Further well-designed clinical trials are required to strengthen the evidence for all of these interventions.
Gutierrez-Castrellon [25] Mexico	RCTs, published between 1960 and 2015 for the treatment of IC	Search between January 1960 and August 2015 in 7 databases and databases of the principal international regulatory agencies English or Spanish language only. Grey literature not searched	Probiotics, Soy, herbal, acupuncture, manipulation, massage	duration of crying after 21 to 28 days of treatment	Yes, alongside Network meta-analysis; No	Yes: Cochrane ROB (not reported for each trial): partial reporting of AEs	Based on systematic analysis of evidence and networking meta-analysis approach use of *L*. *reuteri* DSM17938 seems to be the most evidence-based significant intervention to reduce the duration of crying time in infantile colic. The associated evidence for the use of other interventions such as, herbals, acupuncture, or spinal massage is reduced or significantly biased to let us recommend as potential interventions.
Spinal manipulation							
Dobson [26] UK Cochrane review	RCTs, infants < 6 mths, assessed by clinicians as suffering from colic (all unexplained crying were accepted)	Searched 11 databases, conference proceedings, and trials registries. In addition, CentreWatch, NRR Archive and UKCRN were search in December 2010	Chiropractic, osteopathy or cranial osteopathy alone or in conjunction with other interventions	1. Change in hours crying time per day (post-treatment versus BL) 2. Presence/absence of colic after treatment or FU, or both, that is, the number of infants in which excessive crying resolved (using the definition of those conducting the trial) 3. Any reported AEs, e.g. injury, stroke, arterial dissection, worsening of symptoms	Yes: Yes	Cochrane ROB: Yes	The majority of the included trials appeared to indicate that the parents of infants receiving manipulative therapies reported fewer hours crying/day than parents whose infants did not, based on contemporaneous crying diaries, and this difference was statistically significant. The trials also indicate that a greater proportion of those parents reported improvements that were clinically significant. However, most studies had a high risk of performance bias due to the fact that the assessors (parents) were not blind to who had received the intervention. When combining only those trials with a low risk of such performance bias, the results did not reach statistical significance. Further research is required where those assessing the treatment outcomes do not know whether or not the infant has received a manipulative therapy. There are inadequate data to reach any definitive conclusions about the safety of these interventions.
Gleberzon [27] Canada	Human pptss aged ≤ 18 Involve 2+ ppts, treatments administered by a chiropractor; prospective or retrospective studies, studies using an outcome measure for determining the effect of chiropractic care	2 databases published between January 1980 and March 2011 Papers were written in English and published in peer-reviewed journal	Manual HVLA thrusting spinal manipulations	Effectiveness of SMT on colic (alongside other conditions)	No: No	Sackett 1999 quality grading: Yes	Studies that monitored both subjective and objective outcome measures of relevance to both patients and parents tended to report the most favourable response to SMT, especially among children with asthma. Many studies reviewed suffered from several methodological limitations. Further research is clearly required in this area of chiropractic health care, especially with respect to the clinical effectiveness of SMT on paediatric back pain.
Carnes [28] UK	RCTS, case series, cohorts, service evaluation, qualitative studies. Participants aged 0 mths and 12 mths (infants) when received treatment. Healthy, thriving and not receiving other medical interventions, Wessel criteria	9 databases searched from 1990 (date restriction due to update), including peer networks. Grey literature was searched	Where the manual therapy intervention was delivered in primary care by statutorily registered or regulated professional(s)	Unsettled behaviours (including excessive crying, lack of sleep, displays of distress or discomfort (back arching and drawing up of legs) and difficulty feeding. Experience/satisfaction and global change scores. Adverse events	Yes: No	Cochrane RoB: Yes	Some small benefits were found, but whether these are meaningful to parents remains unclear as does the mechanisms of action. Manual therapy appears relatively safe.
Acupuncture							
Skejeie [29] Norway	Completed RCTs; Wessel criteria (+ modified); no exclusion criteria	9 databases (4 Chinese databases) were searched up to February 2017 alongside 1 trial register	Percutaneous needle acupuncture	Chane from BL crying time at mid and end point, and month FU A 30-min MD in crying time between acupuncture and control was predefined as a clinically important difference	Yes: Yes	Cochrane RoB: Yes	Percutaneous needle acupuncture treatments should not be recommended for infantile colic on a general basis.
HERBAL MEDICINE							
Anheyer [30] Germany	RCTs comparing herbal therapy with no treatment, placebo, or medication in children and adolescents (aged 0–18 years) with GI disorders	3 databases were searched through to July 15, 2016. English and German language only.	Different herbal treatment options (homoeopathic form or Chinese medicine were excluded)	NR	Yes: No	Cochrane RoB: Yes	Because of the limited number of studies, results have to be interpreted carefully. To underpin evidence outlined in this review, more rigorous clinical trials are needed.
Probiotics							
Sung [31] Australia	RCTs, < 3 mths at start of oral probiotic supplementation vs placebo, standard care or no care, any probiotic given to either mothers or infants in both term and preterm infants Wessel criteria of colic	3 databases from 1950 to June 2012 limited to ‘all infants (birth to 23 mths)’ plus 2 trials registers non-English language and unpublished data were excluded	Probiotics	Infant crying, measured as the duration or number of episodes of infant crying/distress, or diagnosis of infant colic (Wessel criteria)	Yes: No	Cochrane RoB: No	Although *L*. *reuteri* may be effective as treatment for crying in exclusively breastfed infants with colic, there is still insufficient evidence to support probiotic use to manage colic, especially in formula-fed infants, or to prevent infant crying. Results from larger rigorously designed studies applicable to all crying infants will help draw more definitive conclusions.
Anabrees [32] Saudi Arabia	RCTs or quasi-RCTs, comparing probiotics to placebo, control or other treatment, term healthy infants with colic, < 4 mths old	3 databases plus contacted experts No language restriction (but abstracts needed to be in English)	*L*. reuteri DSM 17938 or *L*. *reuteri* ATCC 55730	treatment success, defined as the % of children who achieved a reduction in the daily average crying time > 50%.	Yes: Yes	Cochrane RoB: No	Although *L*. reuteri may be effective as a treatment strategy for crying in exclusively breastfed infants with colic, the evidence supporting probiotic use for the treatment of infant colic or crying in formula-fed infants remains unresolved. Results from larger rigorously designed studies will help draw more definitive conclusions.
Urbanska [33] Poland	RCTs, children aged 0 to 18 years, trials to compare *L*. *reuteri* DSM 17938 with placebo or no intervention, not just colic included	2 databases searched to April 2014, 2 trials registers No language restrictions. Grey literature not searched	*L*. *reuteri* DSM 17938	NR	Yes: Yes	Cochrane RoB: Yes	Our results precisely define current evidence on the effects of the administration of *L*. *reuteri* DSM 17938 to the paediatric population.
Xu [34] China	RCTs; Aged 3–6 mths Colic diagnosis (Wessel’s criteria) Not excluded if infants have allergy to milk protein or a family history of allergy	7 databases were searched to May 2015. Conference abstracts excluded.	*L*. *reuteri* DSM 17938	Treatment effectiveness (defined as % of children achieving a ≥ 50% reduction in daily average crying time); duration of crying (min/day)	Yes: Yes	Cochrane RoB: Yes	*Lactobacillus reuteri* possibly increased the effectiveness of treatment for infantile colic and decreased crying time at two to three weeks without causing adverse events. However, these protective roles are usurped by gradual physiological improvements. The study is limited by the heterogeneity of the trials and should be considered with caution. Higher quality, multicenter randomised controlled trials with larger samples are needed.
Schreck Bird [35] USA	RCTs Any probiotic compared to placebo or simethicone Administered to term infant with colic	4 databases from 1947 to December 2014. English language & published trials only.	*L*. reuteri DSM 17938 or *L*. *reuteri* ATCC 55730	assessing crying or fussing time	Yes: No	Cochrane RoB: Yes	Supplementation with the probiotic *L*. *reuteri* in breastfed infants appears to be safe and effective for the management of infantile colic. Further research is needed to determine the role of probiotics in infants who are formula-fed.
Dryl [36] Poland	RCTs, efficacy of probiotics (any well-defined strain) compared with placebo	2 databases searched up to April 2016. Grey literature not searched	Probiotics (any well-defined strain)	Treatment success The duration of crying at the end of the intervention	Yes: Yes	Cochrane RoB: No	Some probiotics, primarily *L*. *reuteri* DSM 17938, may be considered for the management of infantile colic. Data on other probiotics are limited.
Sung [37] Australia	DB RCTs (published by June 2017) *L. reuteri* DSM17398 versus a placebo, delivered orally to infants with colic	6 databases and e-abstracts, and clinical trial registries	*L*. *reuteri* DSM17398	Infant crying and/or fussing duration; treatment success at 21 days	Yes (IPD): Yes	Cochrane RoB: No	*L*. *reuteri* DSM17938 is effective and can be recommended for breastfed infants with colic. Its role in formula-fed infants with colic needs further research.

*AEs* adverse events, *BL* baseline, *CAM* complementary and alternative medicine, *DB* double blind, *FU* follow up, *GI*0020gastrointestinal, *HVLA* high velocity low amplitude, *IC* infantile colic, *IPD* individual participant data, *MD* mean difference, *mths* months, *MA* meta-analysis, *NB* to note, *NR* not reported, *ppts* participants, *QoL* quality of life, *RCT* randomised controlled trial, *RoB* risk of bias, *SMT* spinal manipulation therapy, *TAU* treatment as usual, *WL* wait list

We reported the standard mean difference (SMD) and 95% confidence intervals (CI) and results of any tests of heterogeneity reported in the relevant meta-analyses. Individual study results (mean and standard deviation (SD)) of continuous variables and any between-group statistical analysis were reported when there were no pooled results available (Additional file 1: Table S7). Pooled odds ratios or risk ratios and associated 95% CIs were reported for any dichotomous data.

### Data synthesis

Due to the expected overlap of studies and heterogeneity between reviews (particularly with regard to intervention and comparator arms), we conducted a narrative synthesis of the findings rather than pooling of meta-analyses from the included reviews. We applied the following thresholds for the interpretation of the reported *I*^2^ statistic that assesses heterogeneity [18] in any reported meta-analysis.

- 0% to 40%: might not be important
- 30% to 60%: may represent moderate heterogeneity
- 50% to 90%: may represent substantial heterogeneity
- 75% to 100%: considerable heterogeneity

### Assessment of methodological quality/bias of the included reviews

The quality of each systematic review was assessed using the newly developed AMSTAR-2 scale (Assessing the methodological quality of systematic reviews [19]). This is the revision to the validated and frequently used AMSTAR scale [20]. This was used alongside the ROBIS tool (Risk of Bias in Systematic Reviews tool [21]).

Three reviewers (RP, VL, PD) independently assessed each review using both tools. Two reviewers had limited experience of using the ROBIS tool, so a third reviewer, who helped develop the tool (PD), was asked to complete both ratings. Meta-analyses were checked by a statistician experienced in meta-analyses (CP).

### Deviation from the protocol

There were two changes to the protocol; originally, we were going to exclude reviews that reported on multiple conditions of which infantile colic was one, but we changed this to include reviews that had at least two studies of IC described, to capture more data. We also allowed active treatments to be the comparator option.

## Results

### Results of the literature search

**Fig. 1:**
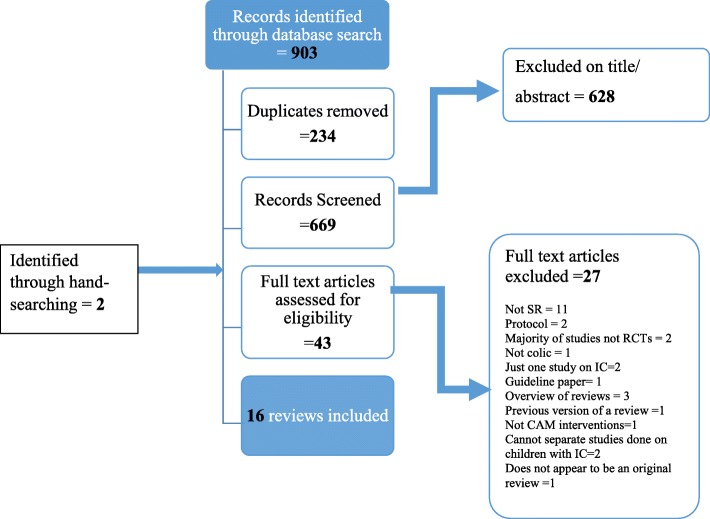
Flow diagram

The search identified 903 potentially relevant papers and 669 titles/abstracts were screened (see Fig. 1). Forty-three full text articles were assessed for eligibility and 16 systematic reviews were included in this overview. Results of the included reviews are presented in Table 1 (details of the individual studies can be found in Additional file 1: Table S7). Only identifiable randomised controlled trials (RCTs) from each review are reported. The summarised AMSTAR-2 scores and ROBIS scores are presented in Tables 2 and 3. Further justification statements for ROBIS are presented in Additional file 1: Table S6. All excluded reviews are listed in Additional file 1: Table S5.

**Table 2:** Results of AMSTAR-2

Author (date) CAM	1. Were PICO components listed?	2. Protocol reported? Any deviations justified?	3. Study design justified?	4. Comprehensive literature search?	5. Was study selection performed in duplicate?	6. Was data extraction performed in duplicate?	7. List of excluded studies? Were these justified?	8. Characteristics of studies provided in detail?	9. Risk of bias assessed?	10. Sources of funding of included studies?	11. Methods used to combine the findings of studies appropriate? Test on heterogeneity?	12. If meta-analysis performed was RoB accounted for?	13. Was RoB discussed in individual studies?	14. Was there discussion of any heterogeneity observed in the results?	15. If a quantitative synthesis, was publication bias investigated and discussed in relation to the results?	16. Reviewers’conflict of interests stated?	Confidence in the review
Multiple CAM therapies																	
Perry [22]	Yes	PY	No	PY	PY	Yes	No	Yes	Yes	No	No MA	No MA	Yes	No	No MA	Yes	Low
Bruyas-Bertholon [23]	No	No	No	No	No	No	No	PY	PY	No	No MA	No MA	No	No	No MA	No	CL
Harb [24]	Yes	No	No	No	Yes	Yes	No	PY	Yes	No	Yes	Yes	Yes	No	Yes	Yes	CL
Gutierrez-Castrellon [25]	Yes	No	No	No	No	No	No	No	No	No	No	No	Yes	No	Yes	No	CL
Manipulation therapies																	
Dobson [26]	Yes	Yes	No	Yes	Yes	Yes	Yes	Yes	Yes	No	Yes	Yes	Yes	No^a^	Yes	Yes	High
Gleberzon [27]	No	No	No	PY	No	Yes	No	PY	No	No	No MA	No MA	Yes	No	No MA	No	CL
Carnes [28]	No	PY	No	PY	Yes	Yes	No	PY	Yes	No	No	No	No	No	No	No	CL
Acupuncture																	
Skejeie [29]	Yes	PY	No	Yes	Yes	Yes	No	Yes	Yes	No	Yes	Yes	Yes	Yes	No	No^d^	Low
Herbal medicines																	
Anheyer [30]	No	No	No	No	No	Yes	No	Yes	Yes	No	No MA	No MA	No	No	No MA	Yes	CL
Probiotics																	
Sung [31]	Yes	No	No	No	Yes	Yes	Yes	PY	Yes	No	Yes	No	Yes	No	No	Yes	CL
Anabrees [32]	Yes	PY	No	PY	No	Yes	No	Yes	Yes	No	Yes	No	Yes	Yes	No	Yes	Low
Urbanska [33]	Yes	No	No	PY	No	No	No	PY	Yes	Yes	No	No	No	No	No	No	CL
Xu [34]	No	No	No	PY	Yes	Yes	No	Yes	Yes	No	Yes	No	No	No	No	Yes	CL
Schreck Bird [35]	Yes	No	No	No	Yes	No	No	Yes	Yes	No	No	No	No	No	No	No	CL
Dryl [36]	Yes	No	No	No	No	No	No	PY	Yes	No	No	No	No	No	No	No	CL
Sung [37]	Yes	PY	No	PY^b^	No	No	No	No	Yes	No	Yes	Yes	Yes	Yes	Yes	No^c^	Low

*CL* critically low, *PY* partial yes, *MA* meta-analysis, *PICO* participants, intervention, comparator, outcomes, *RoB* risk of bias

Grey columns represent the critical domains (see Additional file 1)

^a^Too few studies to perform a test of heterogeneity

^b^Not fully searched and search conducted Dec 2014

^c^Conflict of interest occurred but no indication of how it was dealt with

^d^All included studies were by the author team but did not indicate how this was dealt with

**Table 3:** Tabular presentation for ROBIS results

Review	Phase 2				Phase 3
	1. Study eligibility criteria	2. Identification and selection of studies	3. Data collection and study appraisal	4. Synthesis and findings	5. Risk of bias in the review
Multiple CAM therapies					
1. Perry [22]	Low	Unclear	Low	Low	Low
2. Bruyas-Bertholon [23]	High	High	Unclear	High	High
3. Harb [24]	High	High	Low	High	High
4. Gutierrez-Castrellon [25]	Unclear	High	High	High	High
Manipulation therapies					
5. Dobson [26]	Low	Low	Low	Low	Low
6. Gleberzon [27]	High	High	Unclear	Unclear	High
7. Carnes [28]	Low	Low	Low	High	Unclear
Acupuncture					
8. Skejeie [29]	Low	Low	Low	Low	Unclear
Herbal medicine					
9. Anheyer [30]	Unclear	High	Low	High	High
Probiotics					
10. Sung [31]	Unclear	Low	Low	High	Unclear
11. Anabrees [32]	Low	Low	Low	High	Low
12. Urbanska [33]	Low	High	High	High	High
13. Xu [34]	Unclear	Low	Low	Unclear	Low
14. Shreck Bird [35]	High	High	Low	High	High
15. Dryl [36]	High	High	Unclear	High	High
16. Sung [37]	High	Unclear	Unclear	Unclear	Unclear

The 16 included reviews [22–37] were published between 2011 and 2018. The reviews were conducted from the following countries: three were from the UK and Australia, two from Poland and one each from France, Mexico, Canada, Norway, Germany, Saudi Arabia, China and the USA. The reviews investigated the following therapies: herbal medicine (*n* = 1), acupuncture (*n* = 1), manipulation (*n* = 3), multiple CAMs (*n* = 4) and probiotics (*n* = 7). The majority assessed crying time or reduction in symptoms of colic as the main outcome. There was considerable overlap between studies reported in the included reviews (see Additional file 1: Table S7, for clarity).

## Results from each CAM therapy

### Manipulation reviews

Spinal manipulation was assessed in six reviews [22, 23, 25–28]. Two multiple CAM reviews assessed manipulation but did not pool the results [22, 25]. Both found three trials to be effective [68, 69, 72, 73, or] with the exception of one [71].

Dobson et al.’s [26] Cochrane review of spinal manipulation therapy (SMT) found that five of the six included studies reported a beneficial effect on the course of colic. They found manipulation therapies had a greater effect on daily crying time, reducing it by 1 h 12 min/day on average with moderate heterogeneity (MD = − 1.20; 95% CI − 1.89 to − 0.51, *I*^2^ = 56%). However, there was no evidence of a difference when restricting to the studies judged as having a low risk of performance bias (blinding of parent outcome assessors). Three studies measured full recovery (odds ratio [OR] = 11.12; 95%CI 0.46 to 267.52, *I*^2^ = 89%), but confidence intervals were extremely wide and heterogeneity was substantial. Adverse events were only assessed in one study [83] and none were found. Overall, firm conclusions cannot be drawn from such limited data. The GRADE (Grading of Recommendations Assessment, Development and Evaluation) evaluation indicates low quality of evidence for both outcomes. The results from AMSTAR-2 and ROBIS indicate a high level of confidence in the findings and were judged to be of low risk of bias in all domains.

Gleberzon et al.’s [27] narrative synthesis included three RCTs of SMT for infantile colic [68, 71, 84]. Two of these RCTs were reported in Dobson et al.’s [26] review. Browning et al.’s [84] trial was excluded from the Dobson review as it compared two active treatments (spinal manipulation and occipito-sacral decompression). Just one trial indicated an effect on IC symptoms compared to comparator [68]. There were issues with both quality (AMSTAR-2) and bias (ROBIS) with this review.

Carnes et al. [28], in their update of the Cochrane review by Dobson et al., [26] identified five RCTs of IC. Browning et al.’s [84] trial was included despite comparisons with an active treatment. They conducted a pooled analysis of four colic studies that were also included in Dobson et al.’s meta-analysis [68, 71, 72, 83] (although Carnes et al. used a different version of Miller et al.’s trial [80]). They reported a similar reduction in crying time ((MD = − 1.27 h, 95% CI − 2.29 to − 0.36, *P* = 0.006, *I*^2^ = 69%), again, heterogeneity was substantial and issues with quality and bias were evident.

Gutierrez-Castrellon et al.’s [25] network meta-analysis (NMA) supported the effectiveness of manipulation studies for IC, pooling the results of five RCTs [68, 71, 72, 80, 81] and found a weighted mean difference [WMD] of − 37.4 min (95% CI, − 21.5 to − 67.0), *P* = 0.001, *I*^2^ = 78%) compared to control. The overall confidence in results was critically low (AMSTAR-2) and risk of bias high (ROBIS).

### Herbal medicine

There was one main review of herbal medicine [30]. It assessed various types of herbal medicine for several conditions. We extracted just the data on infantile colic. Evidence was found for different fennel preparations (e.g. oil, tea, herbal compound Colimil) in treating children with colic, whereas peppermint oil was not found to be effective. No serious adverse effects were reported.

Herbal medicine was also assessed in the four multiple CAM reviews [22]. The same studies were included. Gutierrez-Castrellon et al. [25] pooled various herbal extract studies (peppermint, fennel seed oil, Colimil (containing *Matricariae recutita*, *Foeniculum vulgare* and *Melissa officinalis*) in their NMA and demonstrated a weak effect, (WMD = − 61.2 min (95% CI 0.8 to − 122.0, *P* = 0.05, *I*^2^ is 98%)*.* The wide confidence intervals, considerable heterogeneity and the crossing of the line of no effect suggest that herbal medicine has limited effect on crying time. In conclusion, Gutierrez-Castrellon et al. does not recommend herbal medicine for colic.

Harb et al. [24] conducted a subgroup meta-analysis focussing only on extracts containing fennel and demonstrated it to be effective for reducing colic symptoms in solely breast-fed infants. However, this analysis also had considerable heterogeneity and wide confidence intervals (MD = − 72.07, 95% CI − 126.43 to − 17.70, *I*^2^ = 99.5%). It should be noted that much of the fennel oil evidence stems from one trial (Arikan et al. 2008 [73]) which was at particular risk for blinding, selective reporting and issues of randomisation. Massage was one of the arms in this trial; a therapy where it is impossible to blind the therapist. Further, the wide confidence intervals and the crossing of the line of no effect suggest that this trial has limited, if any, value. These issues certainly cast concern on the overall findings.

Perry et al. [22] and Bruyas-Bertholon et al. [23] both reported results that demonstrated fennel herbal extracts to be effective in reducing colic symptoms, but both concluded that the methodological issues of the individual studies (discussed above) call these results into question.

### Acupuncture

One systematic review of acupuncture [29] used individual participant raw data (IPD) which is considered the gold standard of evidence synthesis due to the high level of precision and consistency [38]. A difference was found at the mid time-point analysis favouring acupuncture: MD = − 24.9 min (95% CI − 46.2 to − 3.6; *P* = 0.02, *I*^2^ = 9%), but not after the removal of the one unblinded trial, [47] (MD = − 13.8, 95% CI − 37.5 to 9.9, *P* = 0.25, *I*^2^ = 0%). The GRADE evaluation indicates moderate quality evidence. No major adverse events occurred although acupuncture induced some crying during treatment, believed to be linked to the insertion of needles into the infant [39, 40]. This was a well-conducted review that was judged to be low risk of bias in most domains, but with low confidence in the results (AMSTAR-2). One issue with this review is the authors assessed all their own trials [46, 78, 84], although they appeared to be objective in their evaluation.

Gutierrez-Castrellon et al. [25] pooled two of the above studies [53, 57] and initially found an effect favouring acupuncture (at week 1 and 2) but was no longer evident by week 3 (WMD of − 11.2 min (95% CI 2.0 to − 23.0), *P* = 0.08, *I*^2^ = 0%). There were issues with bias and quality in the review process. Bruyas-Bertholon et al. [23] echoed these findings in their multiple CAM review.

### Probiotics

We identified seven reviews focussing on probiotics [31–37]. The most commonly reported probiotic was *Lactobacillus reuteri* DSM17398. The majority of reviews were rated as having high or unclear risk of bias (ROBIS) and confidence in the results were considered to be critically low (AMSTAR-2).

Sung et al. [31] examined the effectiveness of *L*. *reuteri* DSM17938 versus either placebo or the drug *simethicone.* They meta-analysed three trials of breast-fed infants [15, 62, 74] and found a mean reduction in daily crying time of 67 min compared to control (*N* = 209; MD = − 67.72 [95% CI − 99.79 to − 35.64]) at day 21 with substantial heterogeneity (*I*^2^ = 70%). Anabrees et al. [32] pooled the same three trials but found a mean reduction in crying time of 56 min (*N* = 209:MD = − 56.03; [95% CI − 59.92 to − 52.15], *P* < 0.00001, *I*^2^ = 0%) favouring probiotics, which is a different pooled estimate and *I*^2^ result. This appears to be due to differences in how the authors estimated means + SD from medians. Subgroup analysis of probiotics versus indistinguishable placebo showed a similar reduction in crying time at 21 days (MD = − 55.48 [95% CI − 59.46 to − 51.49], *I*^2^ = 0%). In a separate analysis, probiotics were also shown to improve treatment success at 21 days (RR = 0.06 (95% CI 0.01 to 0.25), *P* < 0.000001, *I*^2^ = 0%).

Urbanska et al. [33] and Xu et al. [34] restricted their analysis to trials of *L*. *reuteri* versus indistinguishable placebo only. Urbanska et al. [33] pooled three trials and found that *L*. *reuteri* DSM17938 reduced crying time by 43 min on day 21, (*N* = 244:MD = − 43.32 [95% CI − 67.62, − 19.02], *P* = 00005). Heterogeneity was substantial (*I*^2^ = 79%). A subgroup analysis of two studies of just breastfed infants was conducted and a greater reduction in crying time of 56 min (*I*^2^ = 0%) was found. Xu et al. [34] meta-analysed five trials and demonstrated that *L. reuteri* decreased crying time at 21 days by 45 min (WMD = − 45.83; [95% CI − 59.45 to − 32.2], *P* = 0.0001). Heterogeneity was moderate (*I*^2^ = 57%). At 4 weeks, pooling three trials demonstrated a similar effect (WMD = − 56.32 min (95% CI − 89.49 to − 23.16, *P* = 0.001; *I*^2^ = 87.7%). They also found that *L*. *reuteri* improved colic treatment effectiveness at three but not 4 weeks.

Schreck Bird et al. [35] pooled five trials of *L*. *reuteri* (ATCC55730 or DSM17938) versus either placebo or *simethicone* and found that there were more responders (infants with ≥ 50% reduction in crying/fussing time from baseline) in the probiotic group than the control (2–3-fold greater chance of responding: RR = 2.34, *P* = 0.01). A fixed-effects model demonstrated substantial heterogeneity (*I*^2^ = 86.1%). Dryl et al. [36] assessed both probiotics (*L. reuteri* DSM17938, *Lactobacillus rhamnosus* GG4.5) and synbiotics and also found a reduction in the average crying /fussing time. Again, responders were defined as above (RR = 1.67[95% CI 1.10 to 2.51]).

The most up-to-date review was by Sung et al. [37] who compared *L. reuteri* DSM17398 versus placebo using individual participant raw data (IPD). Assessments took place at day 7, 14 and 21. The probiotic group averaged less crying and/or fussing time than placebo group at all time points. The results at day 21 using IPD were − 25.4 min (95% CI − 47.3 to − 3.5) *P* < 0.05 (adjusted for baseline). Intervention effects were particularly impressive in breastfed infants—MD = 46.4 min (95% CI − 67.2 to − 25.5, *P* < 0.05), but not so in formula-fed infants—MD = 41.0 (95% CI − 20.1 to 102.2, *P* > 0.05).

Probiotics were also discussed as part of four multiple CAM reviews [22–25]. Both the NMA [25] and Harb et al.’s [24] meta-analysis supports the above findings that *L. reuteri* reduces crying time compared to placebo or *simethicone* (WMD = − 51.3 min (95% CI − 30.5 to − 72.2), *P* = 0.0001, *I*^2^ = 42%; MD = − 55.84, 95% CI − 64.41 to − 47.26), *I*^2^ = 77.1%, displayed respectively). Bruyas-Bertholon et al. [23] also found probiotics to be effective, whereas Perry et al. [22] did not; however, this latter review was conducted prior to the recent supportive evidence.

### Other CAM therapies

Several other CAMs were assessed as part of the multiple CAM reviews [22–25]. Three trials favoured sugar solutions over either placebo control [59, 61], or no treatment [73]. Three trials [66, 73, 82] assessed massage, of which a pooled estimate of two trials [66, 82] demonstrated an effect. One trial on reflexology [67] found no difference compared to the control. Two trials assessed soy added to formula [60, 78], but it was not possible to report the pre-washout data [60] or distinguish the soy data from other supplements analysed.

### Adverse events

With regards to the safety of the reported CAMs, there have been some concerns raised in recent years. In particular, manipulation therapies have come under scrutiny [41, 42] but serious adverse events are rare [43]. Reported adverse events from probiotics are generally mild gastrointestinal disorders such as abdominal cramping, nausea, diarrhoea, flatulence and taste alteration [44].

Poor reporting of adverse events (AEs) is a common criticism of CAM research [45]. It is particularly difficult in infants who cannot communicate their responses effectively. AEs in our included reviews were primarily based on parental reports. However, nine of the 16 reviews did report on AEs with the majority reporting that there were none. The acupuncture review [29] had the highest number of AEs from acupuncture (i.e. bleeding at acupoint, increased hiccoughing), although these are relatively minor. Just six reviews did not report or only partially report on AEs [23, 25, 27, 31, 32, 36]. In addition, several trials ignored safety issues by not providing the reasons why subjects dropped out. Also, due to the small sample sizes of the trials, it is difficult to draw definitive conclusions on safety.

### Quality of the included reviews

#### Results of AMSTAR-2

A summary of the AMSTAR-2 results can be found in Table 2. Most reviews were rated as having critically low confidence in the results, four were rated a low and one (the Cochrane review [26]) was considered to have high confidence in the results. Seven questions that relate to critical domains were identified by Shea et al. [19]; more information about these domains can be found in Additional file 1.

#### Results of ROBIS

The ROBIS tool is divided into four domains (see Table 3 for summary of results and Additional file 1: Table S6 for full results). With regard to domain 1, which assessed any concerns regarding specification of study eligibility criteria, six reviews achieved a low risk of bias rating overall [22, 26, 28, 29, 32, 33]. Domain 2 assessed any concerns regarding methods used to identify/select studies and six achieved a low risk of bias rating overall [26, 28, 29, 31, 32, 34], two were rated as unclear [22, 37] and the remaining eight achieved a high risk of bias rating.

Domain 3 assessed concerns regarding methods used to collect data and appraise studies. Ten of the 16 reviews achieved a low risk of bias rating overall. With regards to domain 4, which assessed concerns regarding the synthesis and findings, the majority were rated as high risk of bias. The final section of the tool provides a rating for the overall risk of bias of all the reviews; four achieved a low rating [22, 26, 32, 34], four [28, 29, 31, 37] an unclear rating and eight a high rating [23–25, 27, 30, 33, 35, 36].

## Discussion

### Summary of the main results

#### Manipulation

From the six reviews that reported on spinal manipulation, the most robust evidence comes from Dobson et al.’s [26] Cochrane review, rated as good quality (AMSTAR-2) with low risk of bias (ROBIS). The other reviews had issues with bias (apart from two [22, 28]) and quality. Most trials indicate a positive effect on crying time alongside other improvements (e.g. ‘recovery from colic’). Blinding was an issue in most trials as the clinician will always know whether they performed a treatment. Thus, the effectivenss of the intervention was called into question when the analyses were restricted to trials with adequate blinding.

#### Herbal medicine

From the five reviews that reported on herbal medicine, preparations containing peppermint demonstrated no evidence of effect. Preparations containing fennel oil demonstrated some effect for reducing the symptoms of colic, but there were limitations to these findings with regards to quality and bias in the review process. Well-designed clinical trials are required to strengthen the evidence.

#### Acupuncture

One systematic review of acupuncture [29] found some evidence of effect of acupuncture but this disappeared when the unblinded trial was removed. They conclude that needle acupuncture should not be recommended for infantile colic on a regular basis. This review scored ‘low’ in most domains of ROBIS, but low overall confidence in result (AMSTAR-2). Gutierrez-Castrellon et al. [25] supported these findings and Bruyas-Bertholon et al. [23] found an initial effect that disappeared at week 3. It is important to note that using acupuncture in infants may raise ethical issues for future trials, as the infant’s response to needle insertion is difficult to evaluate [46] and parents may feel anxious about needles being inserted into their child [40].

#### Probiotics

The majority of probiotic reviews indicated that *L*. *reuteri* is effective in reducing symptoms of colic. Some reviews were of better quality than others as assessed by AMSTAR-2 [32] and ROBIS [32, 34]. There were issues of quality with most of these reviews, so caution is needed before firm conclusions can be drawn. The following issues were identified:

- *Simithicone* was used as a placebo in one trial [62] which was then included in three meta-analyses [31, 32, 37]. As it is an active treatment, the robustness of the results of these meta-analyses needs to be taken with caution.
- Several reviews [33, 34, 36, 37] reported a difference in the efficacy of *L*. *reuteri* between breastfed and formula-fed infants; however, on closer inspection, there was just one study of formula-fed infants which included some mixed-fed babies. Thus, their conclusions over-emphasise the impact of their results.
- Reviewers [35–37] combined different outcomes from the Sung et al. [6] trial. Either ‘crying’ and ‘crying/fussing’ times were analysed, it was unclear why reviewers either combined or separated these outcomes.
- The use of medians in some reported meta-analyses was also problematic and it was unclear if the conversion to means had actually taken place. It was also unclear why different pooled estimates were found when using the same studies [31, 32].

Overall, these reviews suggest that probiotic *L*. *reuteri* may lead to reduced crying time in IC; however, cautious interpretation must be taken due to substantial heterogeneity and small number of trials in the analyses, and low-quality evidence of most of the reviews.

#### Other CAMs

There was some support for both sugar solutions [59, 61, 73] and massage [66, 82], but the trials of reflexology [67] and soy formula [60, 78] did not show support. Concerns regarding the high phytoestrogen content of soy [46, 47] reiterated in a recent Cochrane review of dietary interventions for IC (October 2018) [48] makes this difficult to recommended.

#### Future work

It is important to highlight that the nonspecific effects (e.g. the therapeutic effects of time, attention and touch alongside the placebo effect) of many CAM therapies included in this review are poorly understood but may play a role.

The self-limiting nature of infantile colic means that RCTs are the best way to assess the effectiveness of treatments. Given that there was little convincing evidence for acupuncture, and because funding for CAM research is difficult to obtain, additional research should focus on treatments that offer the most robust evidence. Thus, as encouraging results were demonstrated for manipulation, fennel extracts and sugar solutions, these CAMs require further investigation through well-designed RCTs.

Recent research into colic has focused on probiotics. The majority of reviews concluded that further trials into probiotics for breastfed-only infants are no longer needed. On closer inspection, this conclusion might be premature as the quality of the evidence is currently low. Its role in formula-fed babies certainly requires further research but trialling this will be more problematic as infant formulas commonly contain probiotics [49]. Crying time as measured by parental diaries was the main outcome in most reviews, which is highly subjective; more consideration is needed to accurately measure crying time in future trials (e.g. phone audio or video recordings of the colic episodes).

CAM therapies are difficult to study as some of the most common treatments, (e.g. acupuncture, osteopathy and chiropractic) cannot be adequately blinded. Even trials of other CAMs (such as herbal remedies) have had difficulty in blinding, making it impossible to totally remove bias from the research studies. However, it appears that parents are driving the use of CAM therapies due to limited routine medical care solutions for problem of infant colic. Therefore, those therapies with promising emerging evidence that have, so far, been found safe or without adverse events such as herbal medicines (in particular, fennel oil), probiotics, chiropractic and osteopathic manual therapies may provide reasonable approaches to the problem. Nevertheless, the evidence is far from definitive and more high-quality research is required to help parents decide on the most efficacious therapy for their infant suffering from colic.

#### Potential bias in the overview process

One reviewer (RP) assessed their own work in this overview [22]; however, two other reviewers also assessed each review using AMSTAR-2 and ROBIS. One of the developers of the ROBIS tool was involved (PD). She was invited for her level of expertise in using the ROBIS tool, as the other reviewers had limited experience.

### Strengths and limitations

The search was thorough and included grey literature searching. We believe the systematic approach taken in this overview limits bias. Difficulties in using both ROBIS and some questions on AMSTAR-2 may have led to errors in assessment.

## Conclusions

Spinal manipulation shows promise to alleviate symptoms of colic, although concerns remain as positive effects were only demonstrated when crying was measured by unblinded parent assessors. Fennel is the most promising herbal remedy, but again concerns on the quality of the included studies make any conclusions cautionary.

The majority of the reviews indicate that *L*. *reuteri* DSM17938 should be recommended for breastfed infants with colic, but caution is needed due to the poor quality of the included reviews. Its role in formula-fed babies, in particular, needs further research. Acupuncture and soy are currently not recommended. More rigorous clinical trials are needed for these interventions.

## Supplementary information

**Additional file 1. **Description of included CAMs [51–53, 55]. **Table S4**: table of inclusion/exclusion criteria. Details of the search and data extraction [56]. Table S4 Excluded reviews. Table S6 Summary of the ROBIS domains. **Table S7:** Summary of systematic reviews of CAM treatments for infantile colic. Criteria for assessing confidence in AMSTAR-2.

## Data Availability

Not relevant.

## Abbreviations

AE: Adverse events
AMSTAR-2: Assessing the methodological quality of systematic review
CAM: Complementary and alternative medicine
GRADE: Grading of Recommendations Assessment, Development and Evaluation
IC: Infantile colic
MD: Mean difference
NNT: Number needed to treat
RCT: Randomised controlled trial
ROBIS: Risk of Bias in Systematic Reviews
SD: Standard deviation
SMD: Standard mean difference
TMC: Traditional Chinese medicine
WMD: Weighted mean difference

## References

[1] Lucassen PL, Assendelft WJ, van Eijk JT, Gubbles JW, Douwes AC van Geldrop WJ. (2001). Systematic review of the occurrence of infantile colic in the community. Arch Dis Child.

[2] Vandenplas Y, Abkari A, Bellaiche M (2015). Prevalence and health outcomes of functional gastrointestinal symptoms in infants from birth to 12 months of age. J Pediatr Gastroenterol Nutr.

[3] Benninga MA, Faure C, Hyman PE, St James Roberts I, Schechter NL, Nurko S (2016). Childhood functional gastrointestinal disorders: neonate/toddler. Gastroenterology.

[4] Wessel MA, Cobb JC, Jackson EB, Harris GS, Detwiler AC (1954). Paroxysmal fussing in infancy, sometimes called “colic”. Pediatrics.

[5] Alexandrovich I, Rakovitskaya O, Kolmo E, Sidorova T, Shushunov S (2003). The effect of fennel (foeniculum vulgare) seed oil emulsion in infantile colic: a randomized, placebo-controlled trial. Altern Ther Health Med.

[6] Vik T, Grote V, Escribano J, Socha J, Verduci E, Fritsch M, Carlier C, von Kries R (2009). Koletzko B; European childhood obesity trial study group. Infantile colic, prolonged crying and maternal postnatal depression. Acta Paediatr.

[7] www.nhs.uk/conditions/colic/treatment/ [accessed 10.01.18].

[8] Ernst E, Resch KL, Mills S, Hill R, Mitchell A, Willoughby M, White A (1995). Complementary medicine—a definition. Br J Gen Pract.

[9] Barr RG. Colic and Crying Syndromes in Infants. Pediatrics. 1998;102(Supplement E1):1282-6. 9794970

[10] Terry R, Perry R, Ernst E (2012). An overview of systematic reviews of complementary and alternative medicine for fibromyalgia. Clin Rheumatol.

[11] Ernst E, Canter PH (2006). A systematic review of systematic reviews of spinal manipulation. J R Soc Med.

[12] Posadzki P, Ernst E (2011). Spinal manipulation: an update of a systematic review of systematic reviews. N Z Med J.

[13] Salehi A, Hashemi N, Hadi Imanieh M, Saber M (2015). Chiropractic: is it efficient in treatment of diseases? Review of systematic reviews. Int J Community Based Nurs Midwifery.

[14] Hunt K, Ernst E (2011). The evidence-base for complementary medicine in children: a critical overview of systematic reviews. Arch Dis Child.

[15] Szajewska H, Gyrczuk E, Horvath A (2013). Lactobacillus reuteri DSM 17938 for the management of infantile colic in breastfed infants: a randomized, double-blind, placebo-controlled trial. J Pediatr.

[16] Kianifar H, Ahanchian H, Grover Z (2014). Synbiotic in the management of infantile colic: a randomised controlled trial. J Paediatr Child Health.

[17] Chau K, Lau E, Greenberg S (2015). Probiotics for infantile colic: a randomized, double-blind, placebo-controlled trial investigating Lactobacillus reuteri DSM 17938. J Pediatr.

[18] Deeks JJ, JPT H, Altman DG. Chapter 9: Analysing data and undertaking meta-analyses. In: Higgins JPT, Green S, editors. Cochrane handbook for systematic reviews of interventions version 5.1.0 (updated March 2011): The Cochrane Collaboration. p. 2011. Available from www.cochrane-handbook.org.

[19] Shea BJ, Reeves BC, Wells G, Thuku M, Hamel C, Moran J, Moher D, Tugwell P, Welch V, Kristjansson E, Henry DA (2017). AMSTAR 2: a critical appraisal tool for systematic reviews that include randomised or non-randomised studies of healthcare interventions, or both. BMJ.

[20] Shea BJ, Grimshaw JM, Wells GA, Boers M, Andersson N, Hamel C, Porter AC, Tugwell P, Moher D, Bouter LM (2007). Development of AMSTAR: a measurement tool to assess the methodological quality of systematic reviews. BMC Med Res Methodol.

[21] Whiting P, Savović J, Higgings JP, Caldwell DM, Reeves BC, Shea B, Davies P, Kleijnen J, Churchill R (2016). ROBIS group. ROBIS: a new tool to assess risk of bias in systematic reviews was developed. J Clin Epidemiol.

[22] Perry R, Hunt K, Ernst E (2011). Nutritional supplements and other complementary medicines for infantile colic: a systematic review. Pediatrics.

[23] Bruyas-Bertholo V, Lachaux A, Dubois J-P, Fourneret P, Letrilliart L (2012). Quels traitements pour les coliques du nourrisson. Presse Med.

[24] Harb T, Matsuyama M, David M, Hill RJ (2016). Infant colic—what works: a systematic review of interventions for breast-fed infants. J Pediatr Gastroenterol Nutr.

[25] Gutiérrez-Castrellón P, Indrio F, Bolio-Galvis A (2017). Efficacy of Lactobacillus reuteri DSM 17938 for infantile colic. Systematic review with network meta-analysis. Medicine.

[26] Dobson D, Lucassen PLBJ, Miller JJ, Vlieger AM, Prescott P, Lewith G. Manipulative therapies for infantile colic. Cochrane Database of Systematic Reviews. 2012;(Issue 12. Art. No.: CD004796) 10.1002/14651858.CD004796.pub2. 10.1002/14651858.CD004796.pub2 PMC11665957 23235617

[27] Gleberzon BJ, Arts J, Mei A, McManus EL (2012). The use of spinal manipulative therapy for pediatric health conditions: a systematic review of the literature. J Can Chiropr Assoc.

[28] Carnes D, Plunkett A, Ellwood J (2018). Manual therapy for unsettled, distressed and excessively crying infants: a systematic review and meta-analyses. BMJ Open.

[29] Skjeie H, Skonnord T, Brekke M, Klovning A, Fetveit A, Landgren K, Hallström IK, Brurberg KG (2018). Acupuncture treatments for infantile colic: a systematic review and individual patient data meta-analysis of blinding test validated randomised controlled trials. Scand J Prim Health Care.

[30] Anheyer Dennis, Frawley Jane, Koch Anna Katharina, Lauche Romy, Langhorst Jost, Dobos Gustav, Cramer Holger (2017). Herbal Medicines for Gastrointestinal Disorders in Children and Adolescents: A Systematic Review. Pediatrics.

[31] Sung V, Collett S, de Gooyer T (2013). Probiotics to prevent or treat excessive infant crying. JAMA Pediatr.

[32] Anabrees J, Indrio F, Paes B, AlFaleh K (2013). Probiotics for infantile colic: a systematic review. BMC Pediatr.

[33] Urbanska M, Szajewska H (2014). The efficacy of Lactobacillus reuteri DSM 17938 in infants and children: a review of the current evidence. Eur J Pediatr.

[34] Xu M, Wang J, Wang N, Sun F, Wang L, Liu XH (2015). The efficacy and safety of the probiotic bacterium Lactobacillus reuteri DSM 17938 for infantile colic: a meta-analysis of randomized controlled trials. PLoS One.

[35] Schreck Bird A, Gregory PJ, Jalloh MA, Risoldi Cochrane Z, Hein DJ (2017). Probiotics for the treatment of infantile colic: a systematic review. J Pharm Pract.

[36] Dryl R, Szajewska H (2018). Probiotics for management of infantile colic: a systematic review of randomized controlled trials. Arch Med Sci.

[37] Sung Valerie, D’Amico Frank, Cabana Michael D., Chau Kim, Koren Gideon, Savino Francesco, Szajewska Hania, Deshpande Girish, Dupont Christophe, Indrio Flavia, Mentula Silja, Partty Anna, Tancredi Daniel (2017). Lactobacillus reuteri to Treat Infant Colic: A Meta-analysis. Pediatrics.

[38] Thomas D, Sanyath R, Benedetti A (2014). Systematic review of methods for individual patient data meta- analysis with binary outcomes. BMC Med Res Methodol.

[39] Jindal V, Ge A, Mansky PJ (2008). Safety and efficacy of acupuncture in children: a review of the evidence. J Pediatr Hematol Oncol.

[40] Skjeie H, Brekke M (2015). ‘Big needles, small bodies’—the absence of acupuncture treatment for infants in contemporary Shanghai: a qualitative study. BMJ Open.

[41] Ernst E (2007). Adverse effects of spinal manipulation: a systematic review. J R Soc Med.

[42] Singh S, Ernst E. Trick or treatment. In: The undeniable facts about complementary medicine. New York: Bantam Press; 2008.

[43] Todd AJ, Carroll MT, Robinson A, Mitchell EK (2015). Adverse events due to chiropractic and other manual therapies for infants and children: a review of the literature. J Manip Physiol Ther.

[44] Hojsak I, Fabiano V, Pop TL, Goulet O (2018). Guidance on the use of probiotics in clinical practice in children with selected clinical conditions and in specific vulnerable groups. Acta Paediatr.

[45] Ernst E (2007). “First do no harm” with complementary and alternative medicine. Trends Pharmacol Sci.

[46] Seltenrich N (2017). Phytoestrogens in soy infant formula: association with DNA methylation in girls has unknown implications. Environ Health Perspect.

[47] Donaldson L (2004). Advice issued on soya-based infant formulas. CMO Update.

[48] Gordon M, Biagioli E, Sorrenti M, Lingua C, Moja L, Banks SSC, Ceratto S, Savino F. Dietary modifications for infantile colic. Cochrane Database Syst Rev. 2018;(Issue 10. Art. No.: CD011029) 10.1002/14651858.CD011029.pub2. 10.1002/14651858.CD011029.pub2 PMC6394439 30306546

[49] Braegger C, Chmielewska A, Decsi T, Kolacek S, Mihatsch W, Moreno L, Pieścik M, Puntis J, Shamir R, Szajewska H, Turck D, van Goudoever J (2011). ESPGHAN committee on nutrition. Supplementation of infant formula with probiotics and/or prebiotics: a systematic review and comment by the ESPGHAN committee on nutrition. J Pediatr Gastroenterol Nutr.

[50] Hemarajata Peera, Versalovic James (2012). Effects of probiotics on gut microbiota: mechanisms of intestinal immunomodulation and neuromodulation. Therapeutic Advances in Gastroenterology.

[51] http://www.nhs.uk/conditions/Osteopathy [accessed 10.09.18].

[52] http://www.nhs.uk/conditions/chiropractic [accessed 10.09.18].

[53] Vincent C, Furham A (1997). Complementary medicine. A Research Perspective. John Wiley & Sons.

[54] White A (2009). Western medical acupuncture: a definition. Acupunct Med.

[55] Shojania KG, Sampson M, Ansari MT, Ji J, Doucette S, Moher D (2007). How quickly do systematic reviews go out of date? A survival analysis. Ann Intern Med.

[56] Jadad AR, Moore RA, Carroll D (1996). Assessing the quality of reports of randomized controlled trials: is blinding necessary?. Control Clin Trials.

[57] Schulz KF, Altman DG, Moher D (2010). C CONSORT 2010 statement: updated guidelines for reporting parallel group randomised trials. BMJ.

[58] Higgins JPT, Green GS (2008). Cochrane Handbook for Systematic Reviews of Interventions version 5.0.0.

[59] Akçam M, Yilmaz A (2006). Oral hypertonic glucose solution in the treatment of infantile colic. Pediatr Int.

[60] Treem W, Hyams J, Blankschen E, Etienne N, Paule C, Borschel M (1991). Evaluation of the effect of a fiber-enriched formula on infant colic. J Pediatr.

[61] Markestad T (1997). Use of sucrose as a treatment for infant colic. Arch Dis Child.

[62] Savino F, Emanuela P, Palumeri E, Oggero R, Miniero R (2007). Lactobacillus reuteri (American type culture collection strain 55730) versus Simethicone in the treatment of infantile colic: a prospective randomized study. Pediatrics.

[63] Menthula S, Tuure T, Koskenala R, Korpela R, Könönen E (2008). Microbial composition and fecal fermentation end products from colicky infants: a probiotic supplementation pilot. Microb Ecol Health Dis.

[64] Weizman Z, Alkrinawi S, Glodfarb D, Bitran C (1993). Efficacy of herbal tea preparation in infantile colic. J Pediatr.

[65] Savino F, Cresi F, Castagno E, Silvestro L, Oggero R (2005). A randomized double-blind placebo-controlled trial of a standardized extract of Matricariae recutita, Foeniculum vulgare and Melissa officinalis (ColiMil®) in the treatment of breastfed colicky infants. Phytother Res.

[66] Huhtala V., Lehtonen L., Heinonen R., Korvenranta H. (2000). Infant Massage Compared With Crib Vibrator in the Treatment of Colicky Infants. PEDIATRICS.

[67] Bennedbaek O, Viktor J, Carlsen K, Roed H, Vindling H, Lundbye-Christensen S (2001). Infants with colic: a heterogenous group possible to cure? Treatment by pediatric consultation followed by a study of the effect of zone therapy on incurable colic [in Danish]. Ugeskr Laeger.

[68] Wiberg J, Nordsteen J, Nilsson N (1999). The short-term effect of spinal manipulation in the treatment of infantile colic: a randomized controlled trial with a blinded observer. J Manip Physiol Ther.

[69] Mercer C (1999). A study to determine the efficacy of chiropractic spinal adjustments as a treatment protocol in the Management of Infantile Colic [thesis]..

[70] Mercer C, Nook B. The efficacy of chiropractic spinal adjustments as a treatment protocol in the management of infantile colic. In: Presented at: 5th Biennial Congress of the World Federation of Chiropractic. Auckland; 1999. p. 170-1.

[71] Olafsdottir E, Forshei S, Fluge G, Markestad T (2001). Randomized controlled trial of infantile colic treated with chiropractic spinal manipulation. Arch Dis Child.

[72] Hayden C, Mullinger BA (2006). Preliminary assessment of the impact of cranial osteopathy for the relief of infantile colic. Complement Ther Clin Pract.

[73] Arýkan D, Alp H, Gözüm S, Orbak Z, Çifçi E (2008). Effectiveness of massage, sucrose solution, herbal tea or hydrolysed formula in the treatment of infantile colic. J Clin Nurs.

[74] Savino F, Cordisco L, Tarasco V (2010). Lactobacillus reuteri DSM 17938 in infantile colic: a randomized, double-blind, placebo-controlled trial. Pediatrics.

[75] Landgren K, Kvorning N, Hallström I (2010). Acupuncture reduces crying in infants with infantile colic: a randomised, controlled, blind clinical study. Acupunct Med.

[76] Mi GL, Zhao L, Qiao DD (2015). Effectiveness of Lactobacillus reuteri in infantile colic and colicky induced maternal depression: a prospective single blind randomized trial. Antonie Van Leeuwenhoek.

[77] Alves JGB, de Brito RC, Cavalcanti TS (2012). Effectiveness of Mentha piperita in the treatment of infantile colic: a crossover study. Evid Based Complement Alternat Med.

[78] Campbell JPM (1989). Dietary treatment of infant colic: a double-blind study. Journal of the Royal College of General Practitioners.

[79] Skjeie H, Skonnord T, Fetveit A (2013). Acupuncture for infantile colic: a blinding-validated, randomized controlled multicentre trial in general practice. Scand J Prim Health Care.

[80] Miller J, Newell D, Bolton J (2012). Efficacy of chiropractic manual therapy in infant colic: a pragmatic single-blind, randomised controlled trial. J Manip Physiol Ther.

[81] Heber A, Senger U (2003). DIE OSTEOPATHISCHE BEHANDLUNGBEI3– MONATSKOLIK IM VERGLEICHZURKONVENTIONELLENTHERAPIE. Osteopathic treatment of Infantile Colic [Masters thesis].

[82] Çetinkaya B, Başbakkal Z (2012). The effectiveness of aromatherapy massage using lavender oil as a treatment for infantile colic. Int J Nurs Pract.

[83] Miller J, Newell D, Bolton J (2010). Chiropractic manual therapy for the infant with colic crying: a randomised double blind placebo-controlled trial. Report of a floor presentation during European chiropractors’ union convention, London, 13-15 may 2010. Clin Chiropr.

[84] Browning M, Miller J (2008). Comparison of the short-term effects of chiropractic spinal manipulation and occipito-sacral decompression in the treatment of infant colic: a single blinded, randomised, comparison trial. Clin Chiropr.

[85] Landgren K, Hallström I (2017). Effect of minimal acupuncture for infantile colic: a multicentre, three-armed, single- blind, randomised controlled trial [ACU-COL]. Acupunct Med.

[86] Dupont C, Rivero M, Grillon C, Belaroussi N, Kalindjian A, Marin V (2010). Alpha-lactalbumin-enriched and probiotic-supplemented infant formula in infants with colic: growth and gastrointestinal tolerance. Eur J Clin Nutr.

[87] Roos S, Dicksved J, Tarasco V, Locatelli E, Ricceri F, Grandin U (2013). 454 pyrosequencing analysis on faecal samples from a randomized DBPC trial of colicky infants treated with Lactobacillus reuteri DSM 17938. PLoS One.

[88] Pärtty A, Lehtonen L, Kalliomäki M, Salminen S, Isolauri E (2015). Probiotic lactobacillus rhamnosus GG therapy and microbiological programming in infantile colic: a randomized, controlled trial. Pediatr Res.

