# Supplementary material for: An overview of systematic reviews of complementary and alternative therapies for infantile colic
Source: Syst Rev. 2019 Nov 11;8:271. doi: 10.1186/s13643-019-1191-5 (PMC6844054; doi:10.1186/s13643-019-1191-5)
Supplement: Supplementary file 1 — Additional file 1. Description of included CAMs [51–53, 55]. Table S4: table of inclusion/exclusion criteria. Details of the search and data extraction [56]. Table S4 Excluded reviews. Table S6 Summary of the ROBIS domains. Table S7: Summary of systematic reviews of CAM treatments for infantile colic. Criteria for assessing confidence in AMSTAR-2. [file 13643_2019_1191_MOESM1_ESM.docx]

**Appendix 1: Description of included CAMs**

Probiotics claim to target the gut microbiotica and restore the composition of the gut microbiome which may prevent gut inflammation.^51^ This may be why there has been increased interest in probiotic research in relation to colic in recent years. The most commonly used microbiota are species or strains of *Bifidobacterium*, of *Lactobacillus* and of *Saccharomyces.*^44^

Osteopathy is a way of detecting, treating and preventing health problems by moving, stretching and massaging a person’s muscles and joints.^52^ Whereas chiropractic treatment uses less leverage and quicker manipulations than osteopathy, also uses soft tissue massage, exercise, corsets, splints and supports.^53^ Manual therapies are suggested for infantile colic based on the idea that musculoskeletal strains affect levels of comfort, infant feeding and gut motility.^28^

Acupuncture is the insertion of the tips of needles into the skin at specific points to stimulate nerve impulses. It aims to restore balance to enable the chi to free flow around the meridians. Each meridian is associated with a particular organ.^54^ Western medical acupuncture has evolved from Chinese acupuncture and is more about stimulating the nervous system.^55^

Herbal medicine is the use if plant extracts/materials for food medicine and health promotion. As humans we are better adapted to them then synthetic drugs. Medicinal plants have multiple actions; some of which are highly toxic.^54^

**Appendix 2: Table 4: table of inclusion/exclusion criteria**

| **Type of reviews** | all systematic reviews of randomised controlled trials (RCTS) of infantile colic were included. This was based on the concept that RCTs are the best way to assess the effectiveness of treatments of colic due to the self-limiting nature of the condition.  Quasi-experimental studies were included only if they were assessed alongside RCTs and were in the minority. Systematic reviews of quasi-experimental studies are at higher risk of bias due to lack of random assignment, but we did not want to exclude reviews if the majority of included studies were RCTs. All systematic reviews were included with or without a meta-analysis.  The reviews must have reviewed at least one CAM treatment for infantile colic. However, reviews that assessed several treatments including conventional treatments in the same review were considered if they included at least two relevant CAMs. Systematic reviews that assessed infantile colic alongside other conditions were considered as long as they reported on at least two studies of colic. In addition, we only included reviews from 2011 onwards so that we were only focused on the more up-to-date evidence.^56^ |
| --- | --- |
| **Type of participant** | reviews that included RCTs using human subjects diagnosed with infantile colic using standard diagnostic criteria (e.g. WESSEL criteria^3^) were eligible. No restrictions regarding gender, condition duration or intensity were applied. Age was restricted to infants under 1 year. |
| **Type of intervention** | reviews of effects of any CAM therapies were included: e.g. acupuncture, probiotics, homoeopathy, osteopathy, chiropractic, herbal medicine, reflexology and aromatherapy. Reviews that included multiple CAM therapies were also included, as long as the CAM therapies were not used in combination. Reviews of complex systems of combinations of a range of therapeutic modalities such as Traditional Chinese medicine (TCM) were excluded as it would be too difficult to establish the separate effects of the individual aspects of this combined approach. Reviews that only assessed CAM therapies used as an adjunct therapy to conventional medicine were also excluded. |
| **Type of comparator** | placebo, active treatment, no treatment, treatment-as-usual or waitlist control groups were permissible as the comparator. |
| **Type of outcome** | any review that included studies that reported measures of colic severity (e.g. parent-reported crying diaries; questionnaires and parental interviews) |

**Appendix 3: details of the search and data extraction**

The following databases were searched from their inception to September 2018: Medline, Embase and AMED (via Ovid), Web of Science and Central via Cochrane library, using a combination of subject headings (e.g, MeSH) and key word terms (see below for the search strategy). The first 10 pages of google scholar were also searched (28.8.18). Conference abstracts/protocols were searched using Web of Science, and authors were contacted to establish progress of their work (see Table 5 in Appendix 4). OpenGrey was searched for grey literature and PROSPERO for ongoing reviews. Reviews had to be completed to be included.

All titles and abstracts retrieved from the search were assessed for eligibility against the predetermined inclusion criteria by two reviewers (RP, VL). Any review appearing to meet the inclusion criteria based on the abstract was retrieved as a full document. The full-text articles were read in their entirety to assess eligibility by two reviewers (RP, VL) and decisions on inclusion and exclusion recorded (see Fig. 1 for flow diagram). Any disagreements were discussed with a third author (PD). Excluded reviews were recorded alongside the reasons for exclusion (see Table 4 in Appendix 4).

Reference lists of all full-text articles were hand-searched for additional studies. We did not restrict to English language papers, however, we did restrict to reviews published from 2011 in order to get the most up-to-date reviews.^56^ Authors of any abstracts/protocols were contacted to establish the status of their review.

**MEDLINE SEARCH**

1. systematic review.ti,ab.

2. meta-analysis.pt.

3. meta-analysis.ti,ab.

4. systematic literature review.ti,ab.

5. review.pt.

6. evidence synthesis.ti,ab.

7. 1 or 2 or 3 or 4 or 5 or 6

8. homeopathy.ti,ab.

9. homeopathic.ti,ab.

10. homeop*.ti,ab.

11. homoeopathy.ti,ab.

12. homoeopath*.ti,ab.

13. homoop*.ti,ab.

14. exp Homeopathy/

15. acupuncture therapy.ti,ab.

16. electroacupuncture.ti,ab.

17. acupuncture*.ti,ab.

18. acupoint.ti,ab.

19. meridian.ti,ab.

20. moxibustion.ti,ab.

21. exp acupuncture/

22. (spin* adj3 manipulation*).ti,ab.

23. (osteopath* adj manipul*).ti,ab.

24. (high adj3 velocit* thrust).ti,ab.

25. (spin* adj3 adjust*).ti,ab.

26. (sham adj3 manipulation*).ti,ab.

27. exp Manipulation, Chiropractic/

28. exp Manipulation, Spinal/

29. exp Manipulation, Osteopathic/

30. chiropract*.ti,ab.

31. osteopath*.ti,ab.

32. exp Hypnosis/

33. (hypno* or autogenic* or mesmer* or guided ima*).ti,ab.

34. reflexolog*.ti,ab.

35. reflexolog* treatment*.ti,ab.

36. foot massage*.ti,ab.

37. zone therap*.ti,ab.

38. (herbal* or medical herbal*).ti,ab.

39. exp Drugs, Chinese Herbal/

40. exp Phytotherapy/

41. probiotics.ti,ab.

42. exp probiotics/

43. L reuteri.ti,ab.

44. 8 or 9 or 10 or 11 or 12 or 13 or 14 or 15 or 16 or 17 or 18 or 19 or 20 or 21 or 22 or 23 or 24 or 25 or 26 or 27 or 28 or 29 or 30 or 31 or 32 or 33 or 34 or 35 or 36 or 37 or 38 or 39 or 40 or 41 or 42 or 43

45. infant*.ti,ab.

46. (neonat* or newborn*).ti,ab.

47. (pediatric* or paediatric*).ti,ab.

48. exp Infant/

49. exp Pediatrics/

50. 45 or 46 or 47 or 48 or 49

51. colic*.ti,ab.

52. gastrointest* cramp*.ti,ab.

53. cry*.ti,ab.

54. gastrointest* dysregulation.ti,ab.

55. exp Colic/ or exp Infant/

56. exp Crying/

57. milk hypersensitiv*.ti,ab.

58. 51 or 52 or 53 or 54 or 55 or 56 or 57

59. 7 and 44 and 50 and 58

60. limit 59 to yr="2010 -Current" (to ensure capture of all 2011 papers)

**Data extraction**

One reviewer (RP) extracted data using a standardised form and summarised the review characteristics (see Table 1). Extracted data was then checked by another reviewer (VL or PD). Disagreements were resolved through discussion. Information was extracted on author, date of review, country, list of studies included in the individual review, intervention and comparator summary, number of participants, diagnosis criteria, meta-analysis results or summary of main between-group results, whether a sensitivity or subgroup analysis was conducted, risk of bias assessment and adverse events.

**Appendix 4: Table 4- Excluded reviews**

| **Author (date)** | **Reason for exclusion** |
| --- | --- |
| 1. Anonymous 2013 | Not a systematic review (SR) |
| 1. Alcantara 2011 | Majority of studies not RCTs |
| 1. Anderson 2017 | Not an SR |
| 1. Batchelor 2015 | Not a formal SR |
| 1. Barnes 2015 | Not an SR |
| 1. Biagioli 2016 | Not an SR |
| 1. Billimoria 2016 | Not colic |
| 1. Breagger 2011 updated by 2. Skorka 2017 | Cannot separate out the studies done on children with colic |
| 1. Cabana 2016 | Protocol for Sung 2018 |
| 1. Cameron 2017 | Not an SR |
| 1. Halpern 2016 | Not a formal SR |
| 1. Hojsak 2018 | Guideline paper |
| 1. Hunt & Ernst 2011 | Overview of reviews |
| 1. Iacovou 2012 | Dietary interventions (normal dietary supplements) |
| 1. Khurshid 2015 | Not an SR |
| 1. Lucassen 2012 | Previous version of Dobson’s 2012 Cochrane review |
| 1. Martin Suarez 2015 | Not an SR |
| 1. Mugambi 2012 | not focusing on colic – just 1 RCT |
| 1. Posadzki 2011 | Overview of reviews |
| 1. Posadzki 2013 | Just one study of IC included |
| 1. Raith 2013 | Just one study of IC included, mostly on general crying |
| 1. Salehi 2015 | Overview of reviews |
| 1. Snyder 2012 | Not an SR |
| 1. Sung 2014 | Protocol of paper |
| 1. Sung 2015 | Not an SR |
| 1. Shabuj 2017 | Does not appear to be an original review |

**Table 5: Table of reviews in progress**

| **1^st^ author** | **Title** | **Prospero ref.** |
| --- | --- | --- |
| Chau K et al | Clinical efficacy and safety of probiotics in paediatric gastroenterology: an overview of systematic reviews | CRD42016032907 |
| Banks S, et al | Probiotics to prevent infantile colic (Cochrane review) | [CRD42017055060](http://www.crd.york.ac.uk/PROSPERO/display_record.php?ID=CRD42017055060) |
| Praveen V, et al | Oral probiotics for infantile colic [Cochrane Protocol] | CRD42015017694 |
| Lu T, et al | Clinical research evidence of massage therapy in infants: a systematic review | CRD42014007593 |
| Savino 2014 | Dietary modifications for infantile colic (Cochrane Protocol) Published October 2018 Gordon et al^49^ | Not registered |

**Appendix 5: Table 6: Summary of the ROBIS domains**

| **Review** | **1. Study Eligibility criteria** | **2.Identification and selection of studies** | **3. Data collection and study appraisal** | **4. Synthesis and findings** | **5. Risk of bias in the review** |
| --- | --- | --- | --- | --- | --- |
| **Multiple CAM therapies** | | | | | |
| Perry  (2011) | **LOW**: There was mention of a review protocol via author correspondence. Inclusion criteria was clearly laid out. | **UNCLEAR:** a thorough search strategy was provided. Some additional searching took place; reference lists and other reviews were hand-searched. Searches did not include trial registries or conference reports. The review was restricted to published studies. Two reviewers looked at full texts but this was not specifically stated for abstract screening. | **LOW:** Two reviewers independently performed data extraction and risk of bias assessment. Study characteristics were extracted and reported in Table 1.  Risk of bias was assessed using appropriate criteria (Jadad score^57^) with allocation concealment being assessed in addition. | **LOW:** There was heterogeneity thus no meta-analysis was performed. Other reviewers performed meta-analyses with this data but found considerable heterogeneity. Each study was discussed and evaluated in detail and a sufficient narrative synthesis occurred. The results of the risk of bias assessment were reported in full. This narrative review assesses the results appropriately and the conclusion reflects this. | **LOW:** The main concerns arising from this were the potential for missed studies either through not including unpublished papers. The conclusions seem fair in relation to these considerations. Generally cautious conclusions. Limitations discussed, including heterogeneity. |
| Bruyas-Bertholon (2012) | **HIGH:** ambiguous inclusion criteria which might be difficult to replicate. Language restrictions could result in missed studies. | **HIGH:** Exclusion of grey literature and restricted to French and English papers only. Inadequate search of databases. | **HIGH:** Table of characteristics and results were not accessed despite writing to authors. Jadad score^57^ was used for risk of bias assessment which does not fully assess allocation concealment. | **HIGH:** Lack of clarity regarding which outcomes are to be synthesised in this review. Limited exploration of heterogeneity and study quality. | **HIGH:** lack of clarity regarding inclusion criteria, methods and results. In adequate search likely to have missed studies. |
| Harb (2016) | **HIGH:** No mention a study protocol but the inclusion criteria was clearly defined. There were restrictions in eligibility criteria such as date and language. | **HIGH:** Comprehensive search strategy but unpublished/grey literature were not considered. | **LOW**: It was unclear whether two reviewers were involved in the risk of bias assessments but we have assumed so from the text. | **HIGH:** High levels of heterogeneity not adequately explored. Possible selective reporting of results. No meta-analysis of dichotomous data without explanation. Effect size in funnel plot not consistent with MA. | **HIGH:** Some restrictions in the search. |
| Gutierrez-Castrellon 2017 | **UNCLEAR:** No mention of a study protocol but all interventions and comparators were pre-specified.  Restrictions to English and Spanish language only. The methods section describes how dichotomous data will be handled but there is only one continuous outcome described (duration of crying). | **HIGH:** Search strategy appears comprehensive but only 75 articles were retrieved in total. No information as to who carried out study selection. Methods additional to database searching not used. | **HIGH:** There was mention of using the CONSORT checklist^58^ which is not a risk of bias tool, it also referred to risk of bias in the results but there was no clear display of this. There was insufficient information about which outcome data were collected or the characteristics of included studies. | **HIGH:** they reported medians in the paper but say they extracted and analysed means. There was significant heterogeneity in the pairwise comparisons that is not explored. Some concerns about clumping different interventions within the same treatment node. Insufficient information to judge whether network is consistent. | **HIGH:** some concerns that studies might have been missed, consistency of the network is unclear and significant heterogeneity is largely ignored. |
| **MANIPULATION THERAPIES** | | | | | |
| Dobson (2012) | **LOW:** Cochrane reviews are required to have a protocol which is peer-assessed before the review can commence. Detailed inclusion criteria were provided. All deviations explained and seem reasonable (e.g. insufficient studies for formal exploration of heterogeneity or funnel plot). | **LOW:** Extensive list of databases searched, plus conference proceedings and trial registries. Author also contacted experts. No concerns with this domain. | **LOW:** No concerns with this domain. Two reviewers independently performed data extraction and risk of bias assessment. Risk of bias was assessed using appropriate criteria (Cochrane RoB^59^). Appropriate study characteristics were extracted and appropriate results were collected. | **LOW:** 6 studies included in the meta-analysis. Forest plot was adjusted for studies with low RoB which changed the conclusion.  There were too few studies to formally conduct sub-group analysis and assess publication bias through funnel plots. | **LOW:** Summary of findings tables and GRADE approach to summarising the evidence are presented. Certainty of the evidence is downgraded due to risk of bias in the included studies and the unexplained heterogeneity. Authors conclusions appear suitably cautious. |
| Gleberzon (2012) | **HIGH:** No mention of a review protocol. Eligibility criteria was ambiguous with regards to suitable comparators and outcomes of interest. Text gives the impression that post-hoc exclusions were made after the searches had been conducted. Grey literature, conference abstracts and foreign language papers were excluded without an acceptable rationale. | **UNCLEAR:** Results were restricted to 2007-2011. The initial yield of 79 articles also seems low (even for a CAM review). It is unclear whether dual screening took place. | **UNCLEAR:** it appears that each author (of 4) reviewed two articles and paired – this adds up to 8 not 16 so this was a bit confusing. Generally, there was a lack of information about which study results were of interest and reported. | **HIGH:** there was no clear explanation why a meta-analysis was not conducted, and the synthesis did not fully incorporate the RoB results. | **HIGH:** Possible bias due to search restrictions (language) were considered but no consideration of publication type mentioned. Only one of outcome (crying) seems to have been reported. Serious concerns identified with regards to specification of eligibility criteria and searching. Reporting of results and synthesis also unclear. |
| Carnes (2017) | **LOW**: Protocol registered on PROSPERO and all aspects reported. | **LOW:** This domain was clear and well- reported. There was a date restriction from 1990 due to “most” research being assessed up to that point were theory-driven position papers. As this was an updated Cochrane review we were less concerned about this restriction. | **LOW:** limited information on participants and intervention/comparator group but the rest of the domain was well addressed. | **HIGH:** Dd not include all possible studies. Heterogeneity was high for reduction in crying time.  Risk of Bias was only mentioned briefly and not linked to results. Both issues were mentioned in the discussion but not explored quantitatively or qualitatively. | **UNCLEAR:** Unclear which of the identified studies actually contributed to the findings of the review. Selective reporting of the results in the results and discussion (some outcomes simply not mentioned e.g. adverse events). |
| **ACUPUNCTURE** | | | | | |
| Skejeie (2018) | **LOW:** Protocol was published on PROSPERO. Restriction was placed on quality criteria. Possible bias to ensure inclusion of their own studies. | **LOW:** No major concerns. Well reported. | **LOW:** No major concerns. Well reported. | **LOW:** but findings not robust from primary outcome. | **UNCLEAR:** we rated this as unclear due to possible conflict of interest in including only studies they were involved in. Otherwise a well-conducted review. |
| **HERBAL MEDICINES** | | | | | |
| Anheyer (2017) | **UNCLEAR:** There was no mention of a protocol There was some degree of transparency but some information missing. No outcomes defined (just effectiveness and safety). This may be due to including multiple conditions. Restricted to English and German only. | **HIGH:** SCOPUS was searched which may have picked up some grey literature. No mention of any other sources. Unclear how many screened the titles/abstracts (but author contribution section implies only one). | **LOW**: No major concerns here. This section was well conducted. | **HIGH:** It was possible some studies were missed, no results displayed. No justification why they only did a qualitative synthesis. Would it have been possible to have pooled jus the fennel studies? Risk of bias was not linked to the results anywhere. | **HIGH:** Lack of clarity on inclusion and possible missing studies which were not highlighted in the discussion. Synthesis and conclusions do not consider risk of bias of the included studies and the conclusions seems a bit over confident. |
| **PROBIOTICS** | | | | | |
| Sung (2013) | **UNCLEAR:** No mention of a review protocol. Criteria was not clear enough regarding the population. Non-English language papers and unpublished data were not included which could cause bias. | **LOW:** No major concerns. Search was thorough and additional searches conducted (ongoing trials and experts) Restrictions based on year of publication were justified. | **LOW:** No concerns with this section. Two reviewers independently performed data extraction and risk of bias assessment. Risk of bias was assessed using appropriate criteria (Cochrane RoB^89^). Appropriate study characteristics were extracted and appropriate results were collected. Misleading presentation of RoB using “YES” instead of “LOW”. It appears that the authors have considered overall attrition rather than differential attrition between arms so possibly haven’t applied the tool correctly. | **HIGH:** It was difficult to establish if there were any departures to pre-defined analysis as there was no protocol.  No funnel plots available. *Simethicone* is treated as being a control rather than an active intervention – is this appropriate when pooling with placebo controls?  Authors pooled medians and seemed to convert medians to means as sensitivity analyses. There's no widely accepted way to pool medians and they do not describe how they converted medians to means. There are some probable holes in their analyses. | **UNCLEAR:** There are concerns as to how decisions were made with regards to which studies were suitable for pooling in a meta-analysis. The use of medians is also problematic.  Choice of outcome measures and timepoints seem to have been made post-hoc and are not adequately justified.  Potential for missed studies due to language restrictions is also a concern. The results do seem suitable cautious however. |
| Anabrees (2013) | **LOW:** registered on PROSPERO – appears to have been adhered to. No concerns. | **LOW:** Additional methods were used to identify relevant reports. Searches seemed fine. Not clear if dual study selection was undertaken. | **LOW:** No major concerns – 2 people conducted risk of bias as noted in author contributions rather than the method section. | **HIGH:** limited information about the analysis plan. Random effects model done not mentioned in protocol (was this post hoc?). Heterogeneity was very high for some outcomes and timepoints but not sure if this was adequately explored. | **LOW:** The main issue in this review were the high levels of heterogeneity, which they do address in their conclusions. |
| Urbanska (2014) | **LOW:** No major concerns although a little limited on information on eligibility criteria. | **HIGH:** Only 2 databases were searched (EMBASE not included). No flow diagram or information on how many records the searched retrieved. Sources additional to bibliographic databases and trial registries were not searched. Single study selection. | **HIGH:** Just one author was used for extraction and assessment, so errors could have been made. | **HIGH:** lack of clarity about the outcomes of interest and analysis plans makes it a difficult domain to assess. Some heterogeneity in the forest plots are ignored. The subgroups (breastfed/formula-fed) were not mutually exclusive. | **HIGH**: Not enough rigor in conducting this review yet conclusions indicated promising results. |
| Xu (2015) | **UNCLEAR:** No mention of a protocol. No inclusion criteria – more like a description of studies identified. No restrictions applied but the flow diagram suggests conference abstracts were excluded. | **LOW:** No major concerns here. | **LOW:** Unclear if one or two people were involved in risk of bias assessment. Generally, no major concerns in this domain. | **HIGH:** The leave-one-out analysis was not predefined.  Heterogeneity was not addressed. Risk of bias as not adequately addressed in relation to results. | **LOW:** High heterogeneity but not conflicting results thus *L reuteri* appears to demonstrate an effect. |
| Schreck Bird (2017) | **HIGH:** no protocol mentioned, and language restrictions imposed. | **HIGH:** searches in each database were sorted by relevance and then a number of these screened. English language restriction applied as a filter. | **HIGH:** Data extraction errors of the Sung et al^7^ trial. | **HIGH: H**ave included *Simethicone* as a placebo (but it is an active treatment). High heterogeneity which was not considered and no consideration of bias when interpreting results. | **HIGH:** Due to restrictions in study selection, inadequate searches and high levels of heterogeneity that were unaddressed we have rated this review as high risk of bias overall. |
| Dryl (2018) | **HIGH:** no protocol mentioned, and language restrictions to English only imposed. | **HIGH:** Unclear how many completed screening of titles/abstracts. Only 2 databases searched. No grey literature searching undertaken. | **UNCLEAR:** No information on whether data extraction or risk of bias assessment was undertaken/checked by a second reviewer. | **HIGH:** High levels of heterogeneity was ignored. No exploration of robustness of studies. Although the authors analysed the data appropriately, the included studies had diverse results and sub-group analyses were largely irrelevant. Future studies are likely to change the pooled estimates. | **HIGH:** emphasised results of breastfed infants but only one study was formula fed (and this had some mixed feeding). Seemed to over-emphasis the significance of this result. |
| Sung (2018) | **HIGH**: Protocol was published separately. One small unpublished study mentioned in the discussion that did not include efficacy data. It mentioned that studies should only be double-blind to be included but this wasn’t the case. | **UNCLEAR:** No mention of who screened the titles/abstracts. Searches only had 282 hits – is this a bit low? No information if study selection was conducted in duplicate. | **UNCLEAR:** No mention whether data extraction or risk of bias assessment was conducted in duplicate. Some study characteristics were reported in table 1. 3 lower quality studies were excluded from the analysis. | **UNCLEAR:** Not clear why sleep duration was reported in 1 study but not put in results. Some outcomes were mentioned in the protocol but not reported on in the results. Funnel plot only carried out on 4 studies and no sensitivity analysis mentioned. Biases were minimal. | **UNCLEAR**: It was unclear whether steps taken to minimise errors in the selection of studies, data extraction and RoB assessment. Possible concern that data as not reported for some outcomes mentioned in the protocol. |

**APPENDIX 6: Table 7: Summary of systematic reviews of CAM treatments for infantile colic (2011 onwards)**

| **Author (date)**  **Country** | **Studies included** | **Intervention Group (IG)**  **as reported** | **Comparator group (CG)**  **as reported** | **No. of participants**  **randomised.** | **Length of intervention:**  **No. of sessions:**  **Follow up (if applicable)** | **Meta-analysis**  **conducted: Y/N**  **Main results as reported (IG V CG)** | **Subgroup/ sensitivity analysis conducted**  **Y/N** | **Risk of Bias assessment/ methodological quality** | **Safety/**  **adverse events mentioned** |
| --- | --- | --- | --- | --- | --- | --- | --- | --- | --- |
| **MULTIPLE CAM THERAPIES** | | | | | | | | | |
| Perry  (2011)  UK^22^ | **Supplements**  1. Akcam 2006^60^  2. Treem 1991^61^  3. Markestad 1997^62^  4. Savino 2007^63^  5. Menthula 2008^64^  **Herbal**  6. Weizman 1993^65^  7. Alexandrovich 2003^4^  8. Savino 2005^66^  **Massage**  9. Huhtala 2000^45^  **Reflexology**  10.Bennedbaek 2001^68^  **Manipulation**  11. Wiberg 1999^69^  12. Mercer 1999^70,71^  13. Olafsdottir 2001^72^  14. Hayden 2006^73^  **Mixed**  15. Arikan 2008^74^ | **Supplements**  1.30% glucose solution: 1 mL  2.Isomil +soy, +polysaccharide  3.2 mL sucrose solution  4.Probiotic *L reuteri* in oil  5. probiotic capsules (*L rhamnosus* & *P freudenreichii*)  **Herbal**  6.Herbal tea 150mL/episode of colic  7.Fennel seed oil in water  8.*Colimil*  **Massage**  9.infant massage  **Reflexology**  10.targeted reflexology  **Manipulation**  11.Chiropractic manipulation (3-5 treatments)  12.Chiropractic manipulation (up to 6 treatments)  13.Chiropractic manipulation for 10 mins 3x  14.Cranial osteopathy (individualized) I hr then 4x 30 mins  **Mixed**  15.a) massage b) fennel tea  c) sucrose solution | **Supplements**  1.Placebo: distilled water  2.Placebo: Isomil only  3.Placebo: distilled water  4. *simethicone*  5.Placebo:cellulose capsules  **Herbal**  6. hot drink  7.Placebo: polysorbate in water  8.Placebo: (same taste, smell, colour, packaging)  **Massage**  9.Crib vibrator  **Reflexology**  10. a) non-targeted reflexology b) TAU  **Manipulation**  11*.Dimithicone* (*Simethicone*) daily + counselling  12.Detuned ultrasound machine  13.Held by nurse for 10 mins 3x (blinded)  14.No treatment  **Mixed**  15.No treatment | (3 crossover)  Total N = 942 | 1. 4 days (assessed at day 4, 8)  2. 9 days (35-day FU)  3. 3-4 days  4. 1x day for 28 days  5. 1x day for 2 wks  6. up to 3x day for 7 days, 14 FU  7. 4x/day for 7 days  8. 2x/evening for 7 days  9. 3x day by mother (after training)  10. 20 mins sessions for 4 days over 2 wks  11. 12-15 days treatment  12. 2 wks  13. 8 days, 8-14-day FU  14. 1x week for 4 wks  15. 2-3x day for 1 wk | NO:  1) 64% V 48% improvement in IG P=0.031 (parental assessment)  2) no between gp diff. in fussing/crying (Pre-washout NR)  3) sig. reduction in colic Sx in IG (parental assessment)  4) sig. reduction in crying time in IG  5) no between gp diff. in total crying time  6) colic improvement sig. better in IG but no diffs in night wakings  7) sig. improvement in colic symptoms in IG;  sig. reduction in hrs of crying/wk (parental diaries)  8) sig reduction in crying time in IG at 7 and 21 days  9) no diff. in colicky crying or symptoms at 4 wks; no diff in parental evaluation  10) No diff between targeted and non-targeted reflexology. Targeted reflexology better than TAU.  11) Sig. reduction in crying time CG at week 1 and 2  12) 93% resolution of symptoms in IG and no reoccurrence at 1 mth  13) No diffs between gps  14) A sig. reduction in crying time in IG and increased sleeping time (parental diaries)  15) A sig. reduction in all groups versus CG | NO | Jadad score^a^  1. 3  2. 4  3. 4  4. 2  5. 3  6. 4  7. 5  8. 3  9. 2  10. 2  11. 1  12. 1  13. 3  14. 2  15. 1 | Yes  1. No AEs  2. NR  3. NR  4. No AEs  5. No AEs  6. No AEs  7. No AEs  8. minor AEs  9. NR  10. NR  11. worsening of Sx in CG  12. NR  13. NR  14. No AEs  15. mentioned in discussion (but no details given) |
| Bruyas-Bertholon (2012)^23^  France  14/31 relevant RCTs reported here | **Non-allopathic drug**  1.Weizman 1993^65^  2. Arikan 2008^74^  3. Alexandrovich 2003^4^  4. Savino 2005^66^  5. Savino 2007^63^  6. Savino 2010^75^  7. Markestad 1997^62^  8. Arikan 2008^74^  9. Akcam 2006^60^  **Manual therapies**  10. Hayden 2006^73^  11. Wiberg 1999^69^  12. Arikan 2008^74^  13. Olafsdottir 2001^72^  14. Landgren 2010^47^  **Soy**  15.Treem 1991^61^ | **Non-allopathic drug**  1.Herbal tea  2.Herbal tea  3.Herbal medicine solution  4.*Colimil*  5.Probiotic *L reuteri*  6.Probiotic *L reuteri*  7*.*Sugar solution 12%  8.Sugar solution 12%  9. Glucose 30%  **Manual therapies**  10.cranial osteopathy  11.SMT  12.SMT  13. SMT  14. Acupuncture  **Soy**  15. soy enriched | Control groups not explicitly reported | **Non-allopathic drug (I:C)**  1.36:36  2.35:35  3.65:60  4.41:47  5.45:45  6.25:25  7.19:19  8.35:35  9.30:30  **Manual therapies (I:C)**  10.14:14  11.25:25  12.35:35  13.50:50  14.46:44  **Soy**  15. 33:33 | 1. 7 days  2. 7 days  3. 7 days  4. 7 days  5. 28 days  6.21 days  7. 4 days  8.7 days  9.4 days  10. 28 days  11.14 days  12. 7 days  13. 8 days  14. 21 days  15. 9 days | NO:  1.proportion no colic 10% P<0.01  2. IG reduced crying by 109 mins/day P=0.001  3.IG decreased crying by 30 mins/day P<0.01  4. IG decreased crying by 90 mins/day  5. IG decreased crying by 83 mins/day (BF infants) P<0.5 day 21  6. IG decreased daily crying by 55 mins/day (BF infants) P=0.02  7. 58% infants showed improvement P<0.01  8. IG decreased crying by 101 mins/day P=0.001  9.16% infants showed improvement P=0.03  10. IG reduced crying by 60 mins/day P<0.001  11. IG reduced crying by 84 mins/day P=0.04  12.IG reduced crying time by 53 min/day P=0.009  13. IG no effective of SMT over control (-18 mins) NS  14. IG reduced crying by 32 mins/day after 1 wk of treatment (P=0.02) and 24 mins after 2 wks (P=0.01) and 20 mins after 3 wks (P>0.05 NS) – no further improvement  15. IG reduced crying by 36 mins/day (P>0.05 NS) |  | Jaded score  1. 4  2. 1  3. 5  4. 4  5. 3  6. 4  7. 3  8. 1  9. 4  10. 3  11. 3  12. 1  13. 4  14. 1  15. 4 | partial  1. No AEs  2. NR  3. NR  4. NR  5. NR  6. NR  7. NR  8. NR  9. NR  10. No AEs  11. No AEs  12. No AEs  13. NR  14. NR |
| Harb  (2016)^24^  Australia  14/17 relevant studies | **Probiotic/symbiotic**  1. Chau 2014^17^  2. Kianifar 2014^16^  3. Mi 2015^76^  4. Savino 2007^63^  5. Savino 2010^75^  6. Sung 2014^6^  7. Szajewska 2013^15^  **Phytotherapies**  8. Akcam 2006^60^  9.Alexandrovich 2003^4^  10. Alves 2012^77^  11. Arikan 2008^74^  12. Markestad 1997^62^  13. Savino 2005^66^  14. Weizman 1993^65^ | **Probiotic/symbiotic**  1.Probiotic *L reuteri*  2.Synbiotic  3.Probiotic *L reuteri*  4.Probiotic *L reuteri*  5.Probiotic *L reuteri*  6.Probiotic *L reuteri*  7.Probiotic *L reuteri*  **Phytotherapies**  8.glucose solution  9.Fennel oil  10. Mint  11. Fennel tea  12. Sucrose solution  13. *Colimil*  14. Herbal tea | **Probiotic/symbiotic**  1.Placebo  2.Placebo  3.Placebo  4.*Simethicone*  5.Placebo  6.Placebo  7.Placebo  **Phytotherapies**  8.Placebo  9.Placebo  10. *Simethicone*  11. No intervention  12. placebo  13. placebo  14. placebo | 1 crossover  **Probiotics**  N completers = 472  Breast fed = 402  **Phytotherapies**  N completers = 418  Breast fed = 350 | 1. 21 days  2.30 days  3.21 days  4.28 days  5.21 days  6.1 month  7.21 days  8. NR  9.7 days  10. 17 days  11. 21 days  12. NR  13. 7 days  14. 14 days | **Probiotics:** YES  Reduction in crying time of 55.9 mins in IG compared to CG (MD=-55.84, 95%CI -64.41, -47.26), I^2^=77.1%  **Phytotherapies:** NO  8. IG 64% v CG 48% of infants improved  9. reduced crying time: response rate* 65% V 24% at day 7  10. reduced crying time: response rate ** 60% V 60% at day 17  11. response rate NR but greater reduction in crying time in IG than CG at day 21 no stats reported  12. response rate***: 63% V 5%  13. crying time: response rate**** 85% V 49% at day 7  14.response rate***** 57% V 26% at day 14 | YES: subgroup meta-analysis on the effects of preparations containing fennel at 21 days | YES: Cochrane ROB  1. Low  2. Low  3. Low  4. Low  5. Low  6. Low  7. Low  8. High  9. Medium  10. Low  11. High  12. Medium  13.Medium  14. Medium | No |
| Gutierrez-Castrellon (2017)^25^  Mexico    NETWORK meta-analysis | **Probiotics**  1.Savino 2010^75^  2.Szajewska 2013^15^  3. Sung 2014^6^  4.Chau 2014^17^  5.Mi 2015^76^  **Soy**  6.Treem 1991^61^  7.Campbell 1989^78^  **Herbal**  8.Alexandrovich 2003^4^  9.Alves 2012^77^  10.Savino 2005^66^  **Acupuncture**  11.Landgren 2010^47^  12.Skjeie 2013^79^  **Manipulation**  13.Wiberg 1999^69^  14.Olafsodottir 2001^72^  15.Miller 2012^80^  16.Hayden 2006^73^  17.Heber 2003^81^  **Massage**  18.Huhtala 2000^67^  19.Çetinkaya 2012^82^ | **Probiotics**  1. *L reuteri* DSM 17938  2. *L reuteri* DSM 17938  3. *L reuteri* DSM 17938  4. *L reuteri* DSM 17938  5. *L reuteri* DSM 17938  **Soy**  **6.** *Isomil* +Soy polysaccaride  **7.** Soy IF  **Herbal**  8. fennel seed oil  9. *Mentha piperite*  10. *colimil*  **Acupuncture**  11. Acupuncture  12. Acupuncture  **Manipulation**  13. SMT  14. SMT  15. Chiropractic manipulation  16. cranial osteopathy  17. systematic osteopathy  **Massage**  18. Massage  19. Massage | **Probiotics**  1. placebo  2. placebo  3. placebo  4. placebo  5. placebo  **Soy**  6. placebo *Isomil* (crossover)  7. standard IF  **Herbal**  8. placebo  9. control  10. control  **Acupuncture**  11. control  12. control  **Manipulation**  13. control  14. control  15. control  16. control  17. control  **Massage**  18. control  19. control | **Probiotics (I:C)**  25: 21  40: 40  67:60  24:28  21:21  **Soy (I:C)**  6. 12: 15  7.11:8  **Herbal (I:C)**  8. 62:69  9. 30:30  10.41:47  **Acupuncture (I:C)**  11.43:38  12. 38:41  **Manipulation (I:C)**  13. 25:20  14.42:33  15.35:34  16.14:14  17.20:20  **Massage (I:C)**  18. 28:30  19. 20:20 | 1.21  2.28  3.21  4.21  5.28  6.14  7.21  8.14  9.7  10.21  11.21  12.28  13.11  14.14  15.14  16.28  17.28  18.21  19.21 | **Comparative efficacy of treatments**  ***L reuteri* DSM 17938** WMD = -51.3 (-30.5 to -72.2), P = 0.0001, I^2^ =42%  **Manipulative** WMD = -37.4 (-21.5 to -67.0), P = 0.001 I^2^ =78%  **Massage** WMD = -37.4 (-2.0 to -78.0), P = 0.04 I^2^ = 0%  **Acupuncture** WMD = -11.2 (2.0 to -23.0), P = 0.08 I^2^ = 0%  **Herbal** WMD = -61.2 (0.8 to -122.0), P = 0.05 I^2^ = 98%  **Soy** – not reported for soy alone | NO | YES: Cochrane ROB  Not reported for each trial  Low for probiotics and moderate for the other RCTs. | 1.No AEs  2-19. NR |
| **MANIPULATION THERAPIES** | | | | | | | | | |
| Dobson (2012)^26^  UK  Cochrane review | 1. Hayden 2006^73^  2. Heber 2003^681^  3. Mercer 1999^70,71^  4. Miller 2010^83^  5. Olafsdottir 2001^72^  6. Wiberg 1999^69^ | 1. cranial osteopathy  2.ostopathy  3. chiropractic treatment  4. a) chiropractic treatment (blinded), b) chiropractic treatment (unblinded)  5.chiropractic treatment  6.chiropractic treatment + advice | 1. no treatment  2. conventional care  3.detuned ultrasound machine (blinded)  4. c) unblinded control:  infants placed on treatment couch but not treated  5. blinded control – held by nurse for same amount of time  6. *dimethicone* (*Simethicone*) daily + advice | Total N = 325 | 1.5 treatments at wkly intervals (4 wks)  2. 4 wkly treatments/visits over a 5-week period  3. max of 6 treatments over 2 wks (FU 1 month later)  4. treatments as needed up to 10 days  5. 3 x over 8 days  6. 3-5 (mean 3.8) treatments over 2 wks | YES:  5 studies (N=231) measured daily hours of crying: manipulative therapies reduced average crying time by 1 hr and 12 mins/day (MD) -1.20; 95% CI -1.89 to -0.51, I^2^ =56%). However, when pooling the low risk of bias studies (parental ’blinding’), the improvement in daily crying hrs was just less than 1 hour (MD -0.57; 95% CI -2.24 to 1.09), I^2^ =75%.  3 studies (N=51) measuring ’full recovery’ from colic (as reported by parents) found manipulative therapies did not result in higher proportions of reported recovery (OR 11.12; 95% CI 0.46 to 267.52), I^2^ =89% | YES:  low risk of selection bias, performance bias and had been peer-reviewed | Cochrane ROB | 1.NR  2. NR  3. NR  4.No AEs  5. NR  6. NR |
| Gleberzon (2012)^27^  Canada  Multiple conditions  assessed including IC | 6 studies of IC (3RCTs)  1. Olafsdottir 2001^72^  2. Browning 2008^84^  3. Wiberg 1999^69^ | 1. SMT  2. SMT  3. SMT | 1.control  2. OSD  3.*dimethicone* (*Simethicone*) | Total N = 170 | 1. 8-14 days FU  2. 4 wks FU  3. 11 days | NO:  1.chiropractic treatment is no more effective than placebo  2. both gps had sig. benefit on IC sx  3. greater decrease in crying in IG compared to CG | NO | Sackett 1999 quality grading  1.37/50  2.41/50  3.37/50 | No AEs reported in any of the trials |
| Carnes (2017)^28^  UK  11 studies on colic: 5 of which were RCTs | 5 RCTs:  1.Browning 2008^84^  2. Hayden 2006^73^  3. Miller 2012^80^  4. Olafsdottir 2001^72^  5. Wiberg 1999^69^ | 1. SMT  2. osteopathic treatment  3. a) chiropractic treatment (blinded), b) chiropractic treatment (unblinded)  4. chiropractic treatment  5. chiropractic treatment | 1. occipital decompression  2. no treatment  3. unblinded control  4. no treatment  5. *dimethicone* (*Simethicone*) |  | 1. 4 wk FU  2. 4 wk FU  3. 10 day FU  4. 8-14 days FU  5. 8-11 days FU | YES:  4 IC studies: (N=191)  Reduction in crying time MD= -1.27 (95% CI: -2.19 to -0.36), P = 0.006, I^2^ =69% | NO | Cochrane ROB:  1. Low  2. high  3. Low  4.Unclear  5.High | The overall risk of reported AEs was low – we could not separate out the colic studies from the other studies. |
| **ACUPUNCTURE** | | | | | | | | | |
| Skejeie (2018)^29^  Norway | 1. Landgren 2010^47^  2. Landgren 2017^85^  3. Skejeie 2013^79^ | 1. standard manual acupuncture  2.a) standard manual acupuncture b) semi-standardised individualised acupoint  3. standard manual acupuncture | 1. identical procedure, no needle insertion  2. identical procedure, no needle insertion  3. identical procedure, no needle insertion |  | 1. 2x/wk for 3 wks  2. 2x/wk for 2 wks  3. 1/day for 3 consecutive days | YES: poled analysis of individual participant data:  No reduction in crying time at end time point. 3 RCTs  MD= -11.4 mins (95%CI: -31.8 to 9.0 I^2^ =0%) at end of treatment  No difference in disappearance in colic symptoms at end of treatment [OR 1.54;95% CI 0.88–2.70]  Heterogeneity was negligible in all analyses. | Yes – sensitivity analyses  of unblinded studies reduces significant result at the mid-point. | Cochrane ROB:  GRADE rating:  moderate quality evidence | 1. minor bleed at acupoint  2. drop of blood on clothes and mark on hand in IG  3.IG: hiccups; regurgitation  CG: small haematoma, restlessness, excessive stools, frequent defecation, light sedation, abdominal pain, unease. |
| **HERBAL MEDICINES** | | | | | | | | | |
| Anheyer (2017)^30^  Germany  Multiple conditions  assessed including IC | 5 studies of IC  1. Arikan 2008^74^  2. Weizman 1993^65^  3. Alexandrovich 2003^4^  4. Savino 2005^66^  5 Alves 2012^77^ | 1. Fennel tea 35 mL up to 150mL  2. herbal tea 150mL  3. 0.1% fennel seed oil  4.*Colimil2mL/kg*  *5.*peppermint oil drops | 1. a) massage b) sucrose solution c) hydrogenised formula  2. placebo tea  3. placebo  4. placebo  5. *Simethicone* | Total N = 491 | 1. 3x day  2. 3s day  3. 7 days of trial and 7 day FU  4. 7 days no treatment, 7 days treatment  5. BL, day 7 and 17 of intervention | 1. IG sig decrease in crying time compared to CG  2. IG superior at eliminating colic & colic improvement compared to CG  3. sig improvement in colic sx & cumulative crying time compared to CG  4. sig reduction in crying time compared to CG  5. no between gp diffs in daily episodes of colic or crying time | No | Cochrane ROB | Yes  1. No AEs  2.No AEs  3. No AEs  4.vomiting, sleepiness and constipation  5. No AEs |
| **PROBIOTICS** | | | | | | | | | |
| Sung (2013)^31^  Australia  Multiple conditions  assessed including IC | 1. Dupont 2010^86^  2. Mentula 2008^64^  3. Savino 2007^63^  4. Savino 2010^75^  5. Szajewska 2013^15^ | 1.*L rhamnosus*, *B infantis,* *α-lactalbumin* in formula  2.*L rhamnosus,* *B breve*, & *P freudenreichii spp shermanii*) capsules  3.*L reuteri* drops (10^8^ cfu/day)  4.*L reuteri* drops (10^8^cfu/day)  5.*L reuteri* drops (10^8^cfu/day) | 1.placebo formula  2.placebo capsules  3.60 mg/d *simethicone*  4.placebo drops  5.placebo drops |  | 1.1 mth  2.2 wks  3.1 mth  4.21 days  5.21 days | YES:  3 studies (N=209) examined the effectiveness of *L. reuteri* v placebo or *simethicone* and found a mean reduction in crying time of 67 minutes per day compared to placebo (MD= -67.72 (-99.79 to -35.64) at day 21. However, there was substantial heterogeneity I^2^=70% | No | Cochrane ROB | No |
| Anabrees  (2013)^32^  Saudi Arabia | 1.Savino 2007^63^  2.Savino 2010^75^  3. Szajewska 2013^15^ | 1. *L reuteri* ATCCS 55730 10^8^ cfu/day  2. *L reuteri* DSM 17938 10^8^ cfu/day (30 mins before feed in am)  3. *L reuteri* DSM 17938 10^8^ cfu/day | 1. *Simethicone*  2.placebo  3. placebo | total N = 220: | 1.28 days  2. 21 days  3. 21 days | YES:  3 studies (N=209) examined the effectiveness of *L. reuteri* v placebo or *simethicone* and found a mean reduction in crying time of 56 minutes (MD = -56.03 95%CI: -59.92 to -52.15) at day 21, I^2^ = 0%  Probiotics improved the treatment success  RR of 0.06 (95%CI 0.01 to 0.25) I^2^ = 0%; NNT = 2 | YES:  Placebo only;  Atopy subgroup analysis | Cochrane ROB | NR – but listed as a secondary outcome |
| Urbanska (2014)^33^  Poland  Multiple conditions assessed;  3 on IC | 1. Savino 2010^75^  2. Szajewska 2013^15^  3. Sung 2014^6^ | 1*. L reuteri* DSM 17938  2. *L reuteri* DSM 17938  3. *L reuteri* DSM 17938 | 1. placebo  2. placebo  3. placebo (maltodextrin) | Total N=244 | 1. 21 days  2. 21 days  3. 1 mth | YES: pooled results of 3 RCTs (n = 244) at 21days: *L reuteri* reduced crying time on day 21  MD= -43.32 (95%CI: -67.62, -19.02), I^2^ = 79% | YES: breastfed only | Cochrane ROB | No adverse events – *L reuteri* was well tolerated. In 2011 the US FDA assessed it as safe to add to infant formula |
| Xu (2015)^34^  China | 1.Savino 2010^75^  2. Szajewska 2013^15^  3.Roos 2013^87^  4. Sung 2014^6^  5.Mi 2015  6. Chau 2015^17^ | 1. *L reuteri* 10^8^ cfu/day  *2. L reuteri* 10^8^ cfu/day  3. *L reuteri* 10^8^ cfu/day  4. *L reuteri* 10^8^ cfu/day  5. *L reuteri* 10^8^ cfu/day  6*. L reuteri* 10^8^ cfu/day | 1.identical placebo  2. identical placebo  3. identical placebo  4. identical placebo  5. identical placebo  6. identical placebo | Total N = 423 | 1.21 days,  2.21 days, FU 28 days  3.21 days  4. 1 mth, FU 6 mths  5.21 days, FU 28 days  6.21 days, | YES:  *L reuteri* decreased crying time at:  21 days WMD = -45.83 95%CI: -59.45 to -32.21, P =0.000. I^2^ = 57.1%  4 weeks WMD = –56.32; 95% CI: –89.49 to –23.16; P = 0.001. I^2^ = 94.3%  *L reuteri* improved colic treatment effectiveness at 3 wks RR=2.33 95%CI: 1.38 to 3.93, P=0.002 but not at 4 wks. | YES: ITT and per-protocol analysis | Cochrane ROB | 1. Rhinitis in IG, eczema, fever, otalgy & reflux in CG  2. No AEs reported  3. NR  4. no AEs reported  5. No AEs reported  6. No AEs reported |
| Schreck Bird  (2017)^35^  USA | 1.Savino 2007^41^  2.Savino 2010^53^  3. Szajewska 2013^15^  4.Sung 2014^6^  5.Chau 2015^17^ | 1.*L reuteri* ATCC 55730 10^8^ fu/d  2*. L reuteri* DSM 17938 10^8^ fu/d  3. *L reuteri* DSM 17938 10^8^ fu/d  4. *L reuteri* DSM 17938 10^8^ fu/d  5. *L reuteri* DSM 17938 10^8^ fu/d | 1. *Simethicone*  2. identical placebo  3. identical placebo  4. identical placebo  5. identical placebo | Total N= 449 completers = 388 | 1.Daily for 28 days  2. Daily for 21 days  3. Daily for 21 days  4. Daily for 21 days  5. Daily for 21 days | YES:  Reduction in the average crying time/fussing****  N = 317: more responders in the probiotic gp than CG (2-3-fold greater chance of responding)  RR = 2.34 P=0.01  NB: Sung 2014 data was incorrectly extracted | NO | Cochrane ROB  1.High  2. Low  3.Low  4.Unclear  5.Low | 1 No AEs reported  2. Rhinitis in IG, eczema, fever, otalgy & reflux in CG  3. No AEs reported  4. No AEs reported  5. No AEs reported |
| Dryl  (2018)^36^ | **Probiotics**  1. Chau 2015^17^  2.Mi 2015^76^  3.Savino 2010^53^  4.Sung 2014^6^  5.Szajewska 2013^15^  6.Pärtty 2015^88^  **Synbiotics**  7.Kianifar 2014^16^ | 1. *L reuteri* DSM 17938 10^8^ fu/d  2. *L reuteri* DSM 17938 10^8^ fu/d  3. *L reuteri* DSM 17938 10^8^ fu/d  4. *L reuteri* DSM 17938 10^8^ fu/d  5. *L reuteri* DSM 17938 10^8^ fu/d  6.*L Rhamnosus* GG 4.5 10^8^ fu/d  7.Synbiotic sachet^b^ | 1. placebo  2. placebo  3.placebo  4. placebo  5. placebo  6.placebo  7.placebo | Total N = 471 | 1.21 days  2.21 days  3.21 days  4.28 days  5.21 days  6.28 days  7.30 days | YES:  Reduction in the average crying time/fussing****  RR = 1.67 (95%CI: 1.10 to 2.51 NNT = 5 CI:4-8) | breastfed only; Formula-fed | Cochrane ROB | No |
| Sung (2018)^37^  Australia | 1. Savino 2010^53^  2. Szajewska 2013^15^  3. Sung 2014^6^  4. Chau 2010^17^ | NR here | NR here | Total N = 345  IPDMA data | NR here | YES:  Day 7: pooled analysis from IPD N = 309  -21.0 (95% CI: -42.0 to -0.05); P< 0.0.5  Day 14: pooled analysis from IPD N = 295 -19.4 (95% CI: -41.1 to 2.3); P>0.05  Day 21: pooled analysis from IPD N = 293 -25.4 (95% CI: -47.3 to -3.5) P<0.05 (adjusted for baseline) | YES: breastfed only;  formula fed | Cochrane ROB | 1. No AEs reported  2. no AEs reported  3. No AEs reported  4. No AEs reported |

am =morning; cfu = colony-forming units; CI - confidence interval; *Colimil* (contains *Matricariae recutita, Foeniculum vulgare & Melissa officinalis);* gp- group; hrs - hours; IC - infantile colic; IPDMA - individual participant data meta-analysis; IPD - individual participant data; IF = infant formula; MD- mean difference; mins - minutes; N- number; OR-odds ratio; PB - placebo controlled; RCT- randomised controlled trial; SM - spinal manipulation; sx – symptoms; TAU - treatment as usual; wk - week

a = additional assessment from CRBAT; CRBAT = Cochrane risk of bias assessment tool;

b =Synbiotic sachet containing *L casei, L Rhamnosus, S thgermophilus, B breve, L acidophilus, B infantis, L bulgaricus* & fructooliogosaccharides

* responders defined by a reduction in cumulative crying to <540 min (9hrs)/week; ** responders defined by mother’s opinion and daily episodes of crying

*** responders defined by parents on scale of 1-5; **** Responders defined by having a reduced crying time ≥ to 50% from baseline

***** responders defined as a colic improvement scale

**Appendix 7: Criteria for assessing confidence in AMSTAR-2 (Shea et al^19^)**

| **Rating overall confidence in the results of the review**   - **High** - *No or one non-critical weakness*: the systematic review provides an accurate and comprehensive summary of the results of the available studies that address the question of interest - **Moderate** - *More than one non-critical weakness**: the systematic review has more than one weakness but no critical flaws. It may provide an accurate summary of the results of the available studies that were included in the review - **Low** - *One critical flaw with or without non-critical weaknesses*: the review has a critical flaw and may not provide an accurate and comprehensive summary of the available studies that address the question of interest - **Critically low** - *More than one critical flaw with or without non-critical weaknesses*: the review has more than one critical flaw and should not be relied on to provide an accurate and comprehensive summary of the available studies - *Multiple non-critical weaknesses may diminish confidence in the review and it may be appropriate to move the overall appraisal down from moderate to low confidence |
| --- |
